# Intensity dependent estimation of noise in microarrays improves detection of differentially expressed genes

**DOI:** 10.1186/1471-2105-11-400

**Published:** 2010-07-27

**Authors:** Amit Zeisel, Amnon Amir, Wolfgang J Köstler, Eytan Domany

**Affiliations:** 1 Department of Physics of Complex Systems, The Weizmann Institute of Science, Rehovot, Israel; 2 Department of Biological Regulation, The Weizmann Institute of Science, Rehovot, Israel

## Abstract

**Background:**

In many microarray experiments, analysis is severely hindered by a major difficulty: the small number of samples for which expression data has been measured. When one searches for differentially expressed genes, the small number of samples gives rise to an inaccurate estimation of the experimental noise. This, in turn, leads to loss of statistical power.

**Results:**

We show that the measurement noise of genes with similar expression levels (intensity) is identically and independently distributed, and that this (intensity dependent) distribution is approximately normal. Our method can be easily adapted and used to test whether these statement hold for data from any particular microarray experiment. We propose a method that provides an accurate estimation of the intensity-dependent variance of the noise distribution, and demonstrate that using this estimation we can detect differential expression with much better statistical power than that of standard t-test, and can compare the noise levels of different experiments and platforms.

**Conclusions:**

When the number of samples is small, the simple method we propose improves significantly the statistical power in identifying differentially expressed genes.

## Background

### Microarrays

DNA microarrays are widely used tools for simultaneous measurement of the expression of thousands of genes. Applications of microarrays include (1) identifying differentially expressed genes between two groups, (2) monitoring typical temporal or spatial profiles of genes and (3) classifying samples on the basis of their gene expression signature. The technical procedure of a typical experiment contains the following steps: (1) mRNA is extracted from the sample cells, (2) mRNA is converted to cDNA, (3) cDNA is amplified and labeled, (4) the labeled cDNA is hybridized to a glass slide containing complementary probes, (5) the slide is scanned by a laser, (6) the image is analyzed using a signal processing algorithm which provides the intensity levels and some quality control information. Two types of microarrays are in common use: two color - in which hybridization is performed on a mixture of (differently labeled) cDNA obtained from two samples; and single color (also known as oligonucleotide chip) - in which each sample is hybridized to a different chip. In this work we focus on single color oligonucleotide microarrays.

### Noise

Similarly to every other measurement technique, microarray measurements include noise. We define noise in a model-independent way: repeating an experiment many times under identical conditions generates a distribution of the measured quantity (e.g. the intensity of a single gene, measured in the same sample). The fluctuating random variable that gives rise to this distribution is "noise", quantified by the standard deviation of this distribution. Characterizing the noise distribution is important for assessing the statistical significance of observed differential expression.

### Differentially expressed genes

When biologists compare the expression levels of a gene between two conditions (A and B), say by real time PCR, they repeat the measurements a few times in each condition. Using these repeats, they can estimate basic properties of the noise distribution - usually the mean and the standard deviation - either directly (as proposed here) or indirectly (as done when a t-test is performed). Without any estimate of the noise it is not possible to assign statistical significance to a discovery of differential expression (i.e. calculate a p-value - the probability to get the measured or larger difference of expression from the random fluctuations of the measurement). Practically implementing this approach when using microarrays is difficult because of two main problems: (1) The high cost of each microarray requires careful design of the number and type of repeats in each condition. An insufficient number of repeats can reduce the statistical power of the experiment, thus lowering the sensitivity to detect differentially expressed genes, while using a large number of repeats is very costly. (2) The high throughput nature of this system enables measurement of thousands of genes simultaneously, while in a typical microarray experiment the desired result is a small subset of genes. This introduces a need for control of false positives (type I errors). In contrast to a single gene experiment, in which classically a p-value of 0.05 suffices, using such a p-value for a microarray (of say 10000 genes) will return ~0.05 × 10000 = 500 genes which result from random noise, masking the true differentially expressed genes we are seeking. To overcome this problem, several methods for multiple testing (e.g. Bonferroni, FDR [1]) can be used. However, they usually pass only genes with a much lower "naive" p-value, and therefore their use calls for applying more sensitive methods to estimate single gene p-values.

### The aim of this work

We present a method that improves the statistical power of testing for differentially expressed genes in experiments with small numbers of repeats (or even no repeats at all). We achieve this by showing that the main factor that governs noise is the intensity (expression level) of a gene. Using this, we estimate the measurement noise on the basis of averaging a rough (single gene based) estimate over a large number of genes with similar intensity (and hence similar noise distribution).

### Literature Survey

The issues of normalization and statistical analysis of microarray results were the subject of many studies [2] motivated by the need to reduce the likelihood of reporting false, noise-generated interpretations of the biological systems at hand. In this work we do not deal with the normalization and low-level processing steps and assume that the data are normalized.

Several papers [3-5] identified intensity as a major factor governing microarray induced noise. Novak et al. [3] studied the reproducibility of replicate microarray experiments by comparing the results of parallel assays done with mRNA probes synthesized from the same mRNA sample. They suggest a linear dependency between the replicates' mean intensity and replicates' standard deviation. Tu et al. [4] take this idea forward, using a systematic experimental design, which enables them to compare samples taken from the same cell line but from a different dish. They try to discriminate between biological and technical noise. They also fit an exponential function to the standard deviation line, and find that the hybridization noise has the greatest contribution to the total measurement noise. As a practical result from these noise characterizations, they propose a procedure for calculating a p-value for each gene based on its mean expression level and its fold change. Another attempt to address this issue was made by Huber et al. [5], who presented a variance stabilization approach. They tried to reduce the dependency between the variance and the mean intensity in order to get a uniform (intensity *independent *) noise distribution. They were able to do so by a two step transformation of the data: a linear transformation followed by a sinh^-1 ^transformation. After applying this transformation a simple fold change cut-off is equivalent to a p-value cut-off.

A widely used method for detection of differentially expressed genes, which takes into account the intensity-dependence of the noise is SAM (Significance Analysis of Microarrays) [6]. In SAM, an intensity corrected statistic *d *is calculated for each gene, and significant genes are then identified using comparison to random permutations of the groups. The intensity-dependent correction is obtained by incorporating a constant additive "fudge factor" *s*_0 _in the denominator of the standard t-statistic. The exact value of *s*_0 _is selected to minimize the dependence of the *d *statistic on the expression level. Thus, the fudge factor serves to reduce the significance of the noisier low-intensity genes (a detailed comparison with SAM is shown later in the paper).

Another approach provided by Nykter et al. [7] was to simulate the whole process of microarray measurements starting from the very low level of spot image analysis. This approach is useful in order to simulate large scale microarray data under realistic conditions in order to test and validate data analysis algorithms. A major comprehensive effort to asses the inter- and intra-platform reproducibility of microarrays was done recently by the Microarray Quality Control (MAQC) project [8]. The MAQC study reports intraplatform consistency and high level of interplatform concordance. The goal of this study was to asses reproducibility and *not *to characterize the noise; this was done in a subsequent study by Klebanov et al. [9], who used the MAQC dataset to quantify the level of noise in Affymetrix microarrays. They suggest that the average level of noise in technical replicates (without biological variability) is quite low, as exemplified by the lack of bias induced by such noise in pairwise correlation coefficient estimation.

A generally accepted way to model microarray noise is as a combination of (intensity independent) additive and multiplicative components. We claim that such a parametrization does not provide a good description of the noise and its complexity. In Additional file 1: Section 1, Figure S1, we provide evidence supporting this claim. In what follows we show that our intensity-dependent parametrization does have enough flexibility to provide an accurate description of the noise distribution.

### Notations

Throughout this work we use the following notations:

*I_i,j _*- is the measured *log*_2 _intensity of a gene *i *on microarray *j*.

*I*_0,*i*, *j *_- is the true underlying (without the microarray induced noise) *log*_2 _intensity level of gene *i *on microarray *j*.

*U *- are random variables used in modeling the microarray induced noise.

 - is the true variance of gene *i*.

 - is an unbiased naive estimator for .

 - is the intensity dependent variance estimator (IDVE) for .

Throughout the paper we assume that the measured (log) intensity can be written as *I *= *I*_0 _+ *U*(*I*_0_), where *U *is the noise component of the signal which we aim to characterize (note: this representation is general enough to allow for multiplicative noise, as discussed below).

In the Results section we show in a more formal way that for Affymetrix expression data the noise distribution is indeed mostly intensity dependent, and thus satisfies our assumptions. The same method can be used for any other type of data to test validity of these assumptions. The main point of this paper is to actually estimate this i.i.d. (independent and identical distributed) intensity dependent noise. Our proposed approach, which is based on a local noise estimation, is presented in the Results section, which contains also several practical applications to the analysis of microarray experiments. We then demonstrate the advantage of using our approach by analysis of simulated data and of expression data from several experiments. We close with a discussion of the issue of experimental design and with some concluding remarks.

## Results

### The noise is mostly intensity dependent; formulation and validation

**Figure 1:**
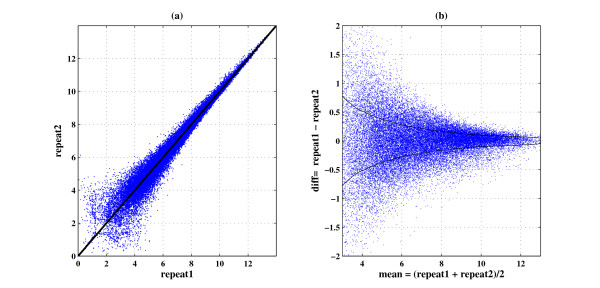
**Noise is intensity dependent**: Typical scatter plot for two experimental repeats. Each dot represents a gene, (a) repeat1 vs. repeat2, the black line is the diagonal (*repeat*1 = *repeat*2), (b) difference vs. mean intensity. The black lines are the running average of the standard deviation estimator (dataset used: GSE19921).

When repeating a microarray experiment under identical conditions (experimental repeats) we expect the true values (the actual expression levels) to be identical, attributing all the differences between repeats to stem from measurement noise. We refer to *experimental repeats *as the scenario where the experiments were repeated with the same type of cell, but grown on different plates, using separate RNA extraction etc., in contrast to purely *technical repeats *which refers to identical RNA being hybridized to two different arrays (some issues related to "biological noise" are discussed in Additional file 1). In most of our analysis we used a publicly available dataset GSE19921 described in detail in the Methods section. Note that if both repeat1 and repeat2 come from the same distribution, their difference is distributed with zero mean and twice the variance. An example of a scatter plot, produced by two technical repeats, is presented in Figure 1a. In the ideal case (zero noise) all the dots should be on the diagonal (*repeat*1 = *repeat*2), but as can be seen, the dots are scattered around the diagonal, with the distance from it reflecting the measurement noise. For an easier estimation of the noise, Figure 1b shows the difference in expression level between the repeats (proportional to the standard deviation) as a function of the intensity - the mean expression of each gene (Figure 1b is obtained from Figure 1a by a rotation of 45°).

**Figure 2:**
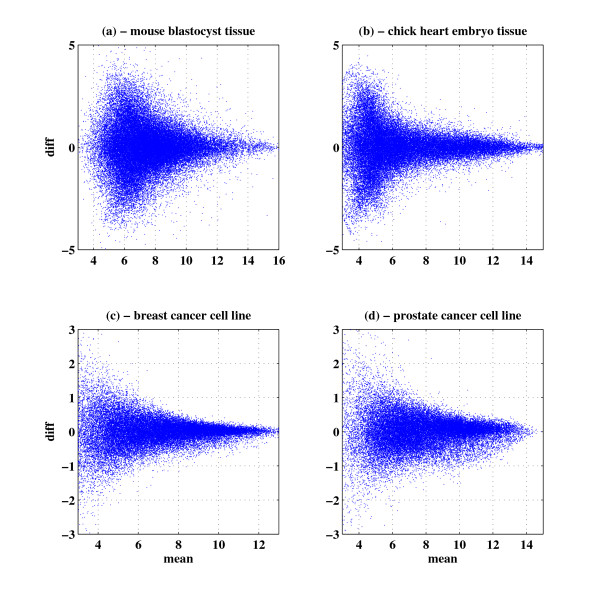
**Intensity dependence appears in many cell and microarray types**: Difference vs. mean scatter plots of experimental repeats from: (a) mouse blastocyst tissue on Affymetrix Mouse 430 array, (b) chick embryo tissue on Affymetrix Chicken Genome Array, (c) breast cancer cell line on Affymetrix HuGene 1.0 ST array, (d) prostate cancer cell line on Affymetrix HuGene 1.0 ST array. All scatter plots show similar intensity dependent noise patterns.

The black lines in Figure 1b present the standard deviation as a function of the intensity. This smooth curve is the result of performing a "running average" of the standard deviation, obtained for each gene *i *by averaging the standard deviations of all genes within a window of intensity ±0.5 centered on *I_i_*. It is evident that the standard deviation of measurement noise decreases with increasing intensity. In Figure 2 we show that this scatter plot pattern appears in many cases, by presenting data for repeats from different tissues and different types of Affymetrix arrays.

In general we can write the measured signal as *I *= *I*_0 _+ *U*(*I*_0_), where *U *is the noise term, that according to the above figures has an intensity dependent distribution. Note that by expressing the measured signal as *I *= *I*_0 _+ *U*(*I*_0_) we are not limiting our noise model to additive noise only; the standard parametrization of the form *I *= *I*_0_*f *+ *a *can be represented by *U*(*I*_0_) = *I*_0_(*f *- 1) + *a*. In fact, by allowing a general intensity-dependent parametrization we allow an even more flexible description than a combination of additive and multiplicative noise terms (see Additional file 1 for more details and fit to data). We show below that the distribution of *U*(*I*_0_) is normal with mean zero and intensity dependent variance. In addition, we claim that the noise terms for different genes are independent random variables, and therefore the noise terms of genes with similar intensities are independent identically distributed random variables.

#### The noise term is normally distributed

**Figure 3:**
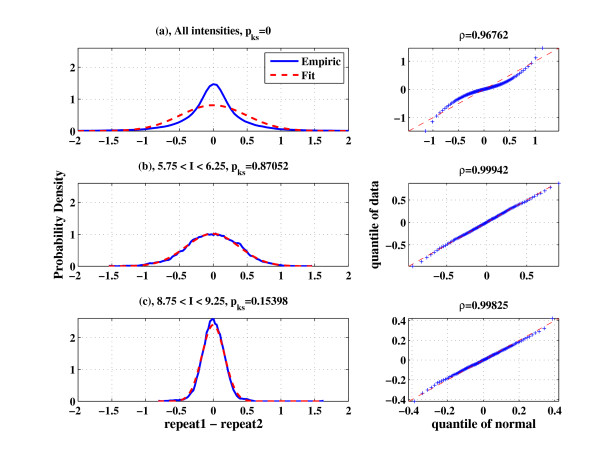
**Genes with similar intensity show normal noise distribution**: Distribution of the differences between experimental repeats (blue line) and their fit to normal distribution (red line). (a) The fit for all the genes on the array is poor, (b) for genes with mean intensity between 5 and 6 and for (c) genes with mean intensity between 7 and 8 excellent fits for normality are obtained (dataset used: GSE19921).

We now turn to study the noise distribution. As a first step, this can be done by looking at the distribution of the difference of two repeats (repeat1-repeat2, as appears in Figure 1b). The difference of two independent random variables (r.v.) with the same mean (*I*_0_) has zero mean and a variance which is the sum of the individual variances; in addition, if the two r.v. have normal distributions, the difference is also normal. While from two duplicates it is hard to infer about the underlying distribution, if the noise term is i.i.d. for a large number of genes one can use measurements from all these genes in order to infer accurately about the distribution. In Figure 3a we plot in blue the experimental probability density function (pdf) of the difference between the repeats over all the genes in the array. The manner in which this smooth pdf is obtained from the data is explained in the Methods section. We also plot (in red) the fitted normal pdf (from estimating the mean and variance). As can be seen, the fit to the normal distribution is very poor. To quantify this observation we performed several normality tests [10,11]; the Kolmogorov-Smirnov (KS) test indeed yielded a very low value (*p_KS _*= 0) and the score from the quantile-quantile (Q-Q) plots with respect to the fitted normal distribution, shown to the right, is also relatively low.

Figures 3b and 3c present similar plots (pdf comparison and KS score on the left and Q-Q plot on the right), but here we use genes that belong to specific bins of mean intensity; Figure 3b is for 5.75 ≤ *I *≤ 6.25 and 3c is for 8.75 ≤ *I *≤ 9.25. Both show good fit to the expected normal distribution (*p_KS _*= 0.87 and *p_KS _*= 0.15 in Kolmogorov-Smirnov test, respectively). The good fit to normal distributions shown in Figures 3b and 3c suggests that the noise term is i.i.d and indeed approximately normally distributed for genes in the same intensity bin. In Additional file 1: Supplemental Figures S2-S6 we provide similar plots for a wide range of intensities and intensity bin sizes, which further support our claim that the normal distribution is a good approximation, and that this holds when the choice of bin size is varied. A second observation is that the variance of the normal distribution changes as we move from one intensity bin to another. This is exactly the reason for the poor fit in Figure 3a, since the distribution of mixed normal random variables with different variances is not normal. These figures also suggest that the noise terms for different genes are independent (note that we claim that the noise terms are independent and not that the gene expression levels are independent). Additionally, the mean of all the experimental pdfs is zero, as expected from data after a normalization procedure which removes biases.

#### The noise variance estimator has a *χ*^2 ^distribution

Expanding this approach to more than two arrays is natural, by plotting the variance estimator as a function of the mean expression level, where the mean and variance estimators for *n *repeats of gene *i *are:(1)

Note that  even in the case of non-normal distribution, since this is the definition of the unbiased variance estimator. If the noise of every gene (in each intensity bin) is normal i.i.d., then the variance estimator should have a chi-square distribution, more accurately:(2)

Therefore:(3)

**Figure 4:**
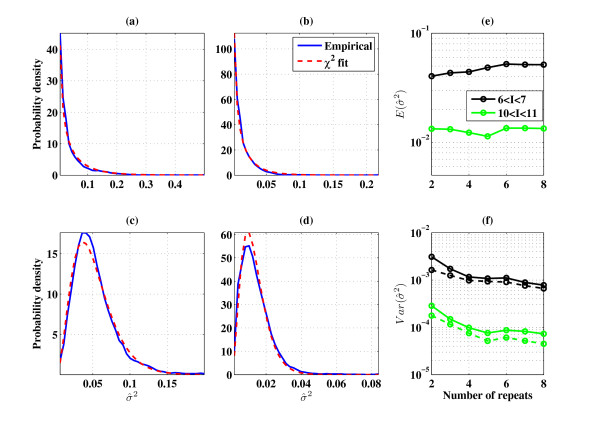
**The noise variance estimator has a *χ*^2 ^distribution**: Empirical probability density function (blue line) of the variance estimator and its fit (red dashed line) to the corresponding *χ*^2 ^distribution (see (2)), in (a) and (c) the mean intensity is between 6 to 7, in (b) and (d) the mean intensity is between 10 to 11. In (a) and (b) we used 2 repeats and in (c) and (d) we used 8 repeats. Panels (e) and (f) show the dependence of the mean (panel (e)) and variance (panel (f)) of  versus the number of repeats at hand, where the black and green lines represent different intensity bins, black for 6 <*I *< 7 and the green for 10 <*I *< 11. The dashed line in panel (f) is the variance expected from (3) (dataset used: GSE19921).

A few points should be kept in mind. The reverse of the statement made above is not correct; we present the agreement between the measured distribution of the variance and the *χ*^2 ^distribution only as supporting evidence and not as a test for normality (which was addressed above). Figure 4 shows the distribution of the variance estimator, where the blue line is the empirical pdf and the red line is the fit expected from the chi-square distribution. The two upper panels (Figures 4a and 4b) are variance estimators based on two repeats and calculated for different mean intensity bins, while in the two lower panels (Figures 4c and 4d) variance is estimated using 8 repeats.

The fit of the distribution of  to the *χ*^2 ^distribution is good. Nevertheless, it should be noted that based only on two repeats, results such as shown in Figures 4a and 4b do not suffice to prove that the noise distribution is i.i.d. The reason is that one can not rule out the possibility of a hypothetical scenario, in which the distribution of variances of the genes is chi-square but in a gene dependent manner (not only intensity dependent and not i.i.d). To rule out this (unlikely) scenario, note that under such a scenario the distribution of  should be independent of the number of repeats, whereas (under our assumptions) we predict dependency of the *χ*^2 ^distribution on the number of repeats. Note that if our predictions are right, in the limit *n *→ ∞ the distribution goes to a delta function at *σ*^2^. We see good fits in Figures 4c and 4d to narrower *χ*^2 ^distributions than those of Figures 4a and 4b. In Figures 4e and 4f we show the dependence of the variance estimator distribution on the number of repeats. As expected, the mean is almost independent of the number of repeats (see Figure 4e), whereas in agreement with eq.(3) the variance decreases (see Figure 4f) as the number of repeats increases. The fact that the distribution of the variance estimator becomes narrower for increasing number of repeats at the same rate as expected by the the chi-square distribution, is consistent with normal i.i.d. distributions of the noise for different genes with similar intensity.

### Estimation of the intensity dependent variance

**Figure 5:**
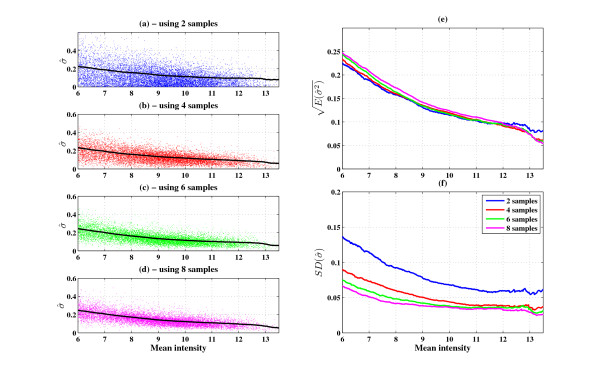
**Comparison of two microarrays suffices to estimate the intensity-dependent noise**: Scatter plots of the square roots of the variance estimators for single genes vs. the mean intensity and its properties for different sample sizes of experimental repeats: (a)-(d) show the scatter and the line estimated by our approach (black line), (e) and (f) shows the average and standard deviation lines (dataset used: GSE19921).

In order to analyze the variance profile, we apply an iterative robust estimator for the intensity dependent variance in constant sized bins (see Methods for details). The result of this estimation for a dataset containing 8 microarrays (Affymetrix HuGene 1.0 ST) is shown in Figure 5. Note that these arrays represent 4 different conditions, with 2 technical repeats in each condition (see Methods section for details). We therefore used only a subset of ~10^4 ^genes which do not have large differences between the conditions (<1.3 fold), but without any constraints about the variation between the duplicates. Hence we view the *n *= 8 microarrays as experimental repeats. Panels a-c of Figure 5 show the properties of this variance estimation using different numbers of repeats (running bin size of 1 in log intensity units). As can be seen, the variance lines are essentially identical (as expected, , see eq. (3)), indicating that a comparison of two microarrays suffices to estimate the intensity-dependent variance of the noise. The variance of this variance estimator indeed decreases as the number of repeats increases, as also expected from eq. (3) (if the noise is only intensity dependent, this variance should converge to zero when the sample size goes to infinity), but the rate of convergence is slow (see also Additional file 1: Supplemental Figure S9). This demonstrates the large fluctuations in the naive variance estimator in such a small sample size. Since there are claims in the literature about a dependency between the noise and the GC content of the probe [12], we show in Additional file 1: Supplemental Figure S7 the variance as a function of the GC content of the probe. As can be seen, the average noise is a flat line suggesting that the noise is *independent *of the GC content.

### The MAQC project dataset

**Figure 6:**
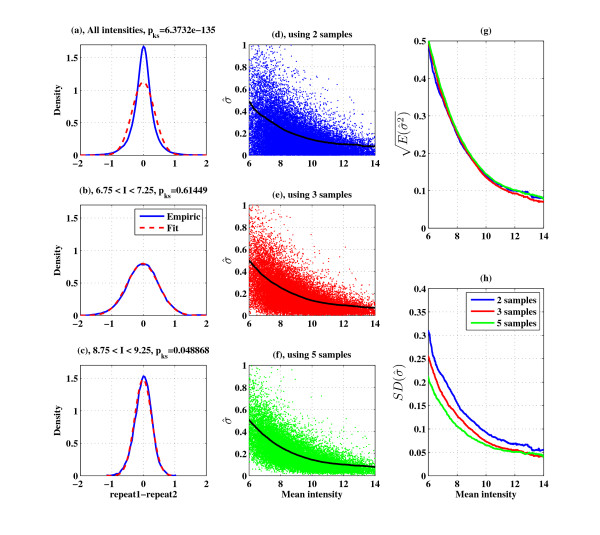
**Technical noise in the MAQC dataset**: (a) - (c) show the empirical pdf of the difference between two technical repeats in blue and the fitted normal pdf in red, for (a) all ranges of intensities, (b) 6.75 <*I *< 7.25, (c) 8.75 <*I *< 9.25. The p-value of the Kolmogorov-Smirnov test for normality is indicated in each figure. (d)-(f) Scatter plots of the square root of the variance estimator vs. the mean intensity, and its properties for different number of repeats; the black line indicates the estimated variance using our approach. (g) and (h) show the average and standard deviation lines from (d)-(f) (data used: GSE5350).

The Microarray Quality Control (MAQC) project [8] was a comprehensive study, aimed at assessing the reproducibility of microarray results across different platforms and within the same platform across different sites. As part of the project, high quality universal human reference RNA (sample A) was hybridized on five identical Affymetrix HG-U133 Plus 2.0 microarrays, all at the same site (Site # 1). The level of noise in such experiments is expected to be the lowest one can achieve, since this is purely technical (hybridization) noise. We used these five replicates to validate our findings regarding the nature of the noise, and to compare the noise levels in these MAQC technical repeats with those of our experimental repeats (the dataset from our Figures 3, 4, 5). As can be seen in Figures 6, the pdf of the difference between two repeats in the MAQC data are quite similar to those of our data (compare to Figures 3a - 3c). Here also, we observe intensity dependent noise distribution; for the intensity bin 7 ± 0.25 the distribution is wider than that of the bin 9 ± 0.25. Here the fit to normal distribution is again poor when we lump all intensities together, but becomes good in specific intensity bins. Figures 6d - 6h are similar to Figures 5a - 5f, and show that estimation of the noise on the basis of two repeats is as good as the estimation based on five repeats, and that the width of the distribution of the variance around its mean value decreases (as predicted by our noise model) as the number of repeats increases. The noise levels of the MAQC data are expected to be different from ours because of the different platform, but since the noise is of the same order as in Figure 5, we can conclude that even for the case of experimental repeats, the dominant contribution to the noise comes from hybridization.

### Implementation

A naive approach for estimating the noise is to calculate the variance estimator for each gene, one at a time, using the *n *repeats in hand (for example, as is implicitly done in a t-test or by using SAM). However, in the case of a small sample size large fluctuations in the variance estimation (used while calculating the *t *statistic) lead to reduced statistical power.

Our proposed method is based on eq. (3); specifically, on the statement that the mean of the variance estimator is the true variance. We use the fact that the noise is intensity dependent, and hence by calculating the mean variance estimator of a (relatively) large number of genes with similar intensity, we get an estimate of the true variance which is significantly improved and stabilized versus the one based on a single gene. Using these intensity dependent standard deviation values, we can assign statistical significance to the differential expression of a gene between two conditions using the more powerful z-test (instead of the t-test or SAM), thus gaining improved statistical power. We demonstrate this improvement on simulated data and on real experimental data.

#### Analyzing data with n ≥ 2 repeats

Consider an experiment with *n *repeats, denoting the expression levels of gene *i *by *I*_*i*, *j *_for *j *= 1, 2⋯*n*. Apply the robust variance estimator procedure outlined in the Methods section to obtain ; where  is the mean expression of gene *i *over *n *repeats, and  is the mean of the naive (*n*-repeat based) single-gene variance estimators, obtained by averaging over the (large number of) genes in the intensity bin of gene *i*. Note that the variance of the quantity . Use this estimate of the noise distribution to find the differentially expressed genes between conditions *A *and *B*, for each of which we have *n *repeats:

1. Calculate .

2. For each gene calculate the statistic .

3. For each gene test the null hypothesis that there is no difference between the expression of the two conditions. Formally stated, the null hypothesis is:(4)

The above suggested p-value calculation is equivalent to using the z-test which provides higher statistical power than the corresponding t-test.

#### Performance on simulated data

**Figure 7:**
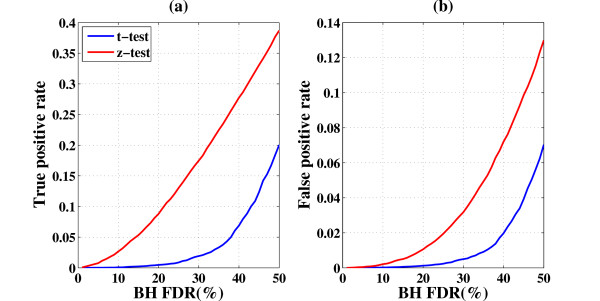
**Increased sensitivity of the proposed method**: Example for the comparison between t-test and z-test (our approach) for the case of 1000 simulated "genes", each with three repeats. 500 out of the 1000 "genes" were truly differentially expressed, (see text for full explanation). For each FDR level the true positive rate (TPR) and the false positive rate (FPR) were calculated based on the ground truth. (a) TPR versus FDR level, (b) FPR versus FDR level. The blue line indicates results of t-test and the red line indicates z-test.

As an example, we simulated a data set containing *i *= 1, 2...1000 "genes", each with *j *= 3 repeats, under two "conditions". In the first condition (denoted as X) all expression values were sampled from a normal distribution with zero mean and unit variance *x_i,j _*~ *N*(O,1) In the second condition (denoted as *Y *) the first 500 genes were sampled from the same distribution, i.e. (y_i,j _~ N(O,1)[i = 1, 2...500]) while the next 500 genes were sampled from a normal distribution with a different mean (y_i,j _~ *N*(*μ*_1_,1)[*i *= 501, 502...1000]) This simulation represents the case when out of the 1000 measured genes, the first 500 are not differentially expressed in conditions X and Y, while the second set of 500 are. Next, for *μ*_1 _= 1 (a relatively weak signal) we calculated the p-values for each gene using (i) the standard t-test and (ii) our suggested method (using z-test). Based on the known true differentially expressed genes we could calculate the true positive rate (TPR) and false positive rate (FPR) for any Benjamini-Hochberg FDR level. This simulation was repeated 100 times, and the results we show are averages over these 100 realizations (the simulation code and output is available as Additional file 2). Figure 7 presents the dependence of the averaged TPR and FPR levels on the FDR used, for both methods. For example at FDR of 10%, the average number of genes passing when the t-test is used to calculate the p-values is 0.5, while 14 genes pass, on the average, using our approach (with FPR of 0.2%). At FDR of 30%, the average number of genes that pass, using t-test, is 10 and 100 genes pass at the same FDR when our approach is used (with FPR of 3%). The results of these figures are also summarized in Additional file 1: Supplemental Figure S8, where the ROC curve is shown for this same value of *μ*_1 _= 1 as well as for a strong signal (*μ*_1 _= 3). The ROC curves clearly demonstrate the superior performance of our z-test - we gain in TPR without over-polluting the discoveries significantly by false positives.

#### Performance on an experimental dataset

**Figure 8:**
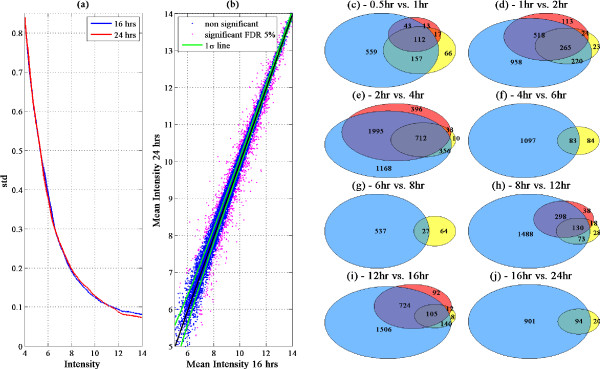
**Application for time course experiment and comparison to other methods**: (a) Estimated standard deviation vs. intensity lines derived from two repeats of data from *t *= 16 hrs and from *t *= 24 hrs after LPS stimulus. (b) Scatter plot of mean intensities measured at *t *= 16 hrs vs. *t *= 24 hrs. Blue dots denote genes that were found as non-significantly differentiating (by our method); magenta - differentially expressed genes, that passed at FDR of 5%; green line - 1 SD. (c)-(j) Venn diagrams of the three groups of genes identified as differentially expressed at 5% FDR in each comparison of consecutive time points by: 2 fold (yellow), SAM (red), and our method (blue), (data used: GSE17721). Note that t-test is not part of the comparison since significantly differentiating genes were found at this FDR only in the 2 hr vs. 4 hr comparison.

In order to test the power gained by using our proposed method on real data, we studied the experimental dataset of Amit et al. [13]. This study includes a time course experiment of gene expression following LPS stimulation of primary mouse bone marrow dendritic cells, using the Affymetrix HT-MG-430A mouse gene array. The experiments we analyzed consisted of 9 time points spanning the first 24 hours following the stimulation, with 2 repeats of each time point. We used these data (following MAS5 normalization) to identify differentially expressed genes between consecutive time points. To this end, we applied several techniques: (1) the standard t-test, (2) the SAM algorithm [6], (3) passing a threshold of 2-fold change, (4) our proposed intensity dependent z-test based method discussed above (with *n *= 2 repeats). As an example we show, in Figure 8a, the intensity-dependent noise profiles measured at two time points (16 and 24 hours following stimulation), estimated from the two replicates. As can be seen, the noise dependent intensity profiles derived at the two time points are similar, with the noise standard deviation decreasing from ~0.8 (at average expression level 4) to ~0.1 (at average expression levels ≥12). Using this noise profile, we apply the z-test (as described above), with a FDR level of 5%. A scatter plot comparison of these two time points (16 vs 24 hrs) is shown in Figure 8b. Genes which are identified as differentially expressed are marked in magenta. As can be seen, 995 candidate genes pass at FDR of 5%. This is in sharp contrast to detection of differentiating genes using the standard t-test or SAM, both of which fail to identify even a single candidate gene at this FDR level.

Figures 8c-8j summarize the number of candidate genes identified by each method (SAM, 2fold, our method) and their overlaps between all consecutive time points using a FDR of 5% (Benjamini Hochberg procedure [1]). As can be seen, utilizing the intensity-dependent standard deviation yields a highly improved statistical power compared to the two alternative methods (SAM and 2-fold change cutoff) as well as the standard t-test (which identifies differentially expressed genes only in the 2-4 hours comparison). An obvious question that arises is the fraction of true and false positives among the large number of differentiating genes found by our method. An indirect way to test this is to consider whether these differentiating genes make "biological sense". To this end, a gene ontology term enrichment analysis was performed on the differentially expressed genes identified by the z-test method using the DAVID web tool [14]. For all consecutive time points tested, a large and significant enrichment is observed in immune response related genes, such as: cytokine production, cell death, phagocytosis and the mitochondrial electron transport chain (Additional file 1: Supplemental Tables S1-S3). Enrichment for immune system related terms is also observed when testing only genes uniquely identified by our method and not by the alternative SAM or 2-fold methods (Additional file 1: Supplemental Tables S4-S6), while we did not observe such enrichment when testing the lists of genes that were identified uniquely either by SAM or by 2-fold change (Additional file 1: Supplemental Tables S7-S8).

A more direct way to test whether the genes discovered by our method and not by SAM are true positives is by an independent experimental validation. We repeated the experiment of Amit et al [13] and tested, by qPCR measurements, the expression profiles of 15 selected genes that were found to be differentially expressed by our method (five of which were not found by SAM or t-test), and compared their time-dependent profiles to those obtained by Amit et al using microarrays. Indeed, although the experiment was done on different murine bone marrow dendritic cells by different people at different labs at a different time, the qPCR profiles of the genes looks very close to those derived from the the array-based profiles (Additional file 1: Supplemental Figure S10). In particular, note that we validated the variation of those five genes that were identified by our method as significantly varying and not identified as such by either SAM or t-test.

#### Analyzing data without repeats

We now turn to the case of a single microarray in each biological condition, without repeats. The only way one can estimate noise in such a case is by viewing the two different conditions as two repeats. Of course, one has to address the question: is it possible to distinguish between differences due to noise, and the true condition dependent changes in gene expression, even when there are no real repeats? Clearly, this scenario is not recommended and one should try to avoid it. Since the technical noise depends mainly on the mean expression level while the real biological variation can be gene specific, naive averaging over the variance estimator () in an intensity bin will produce over-estimation of the variance, because it treats real biological differences also as noise. Under the assumption that the number of genes whose expression levels significantly differ between the two conditions is small, the Robust Variance Estimation procedure described in the Methods section can partially overcome this problem. In this case, we suggest as a heuristic to find for each sample the one or two closest samples (by PCA or any other distance measure), treat these as repeats and apply the Robust Variance Estimation procedure.

#### Experimental design considerations

When performing a DNA microarray experiment, we need to select the number and type of repeats at each condition. While having more repeats gives a higher sensitivity, they cost more. We provide a rough estimate of the required number of repeats in a typical experiment for detecting differentially expressed genes between two conditions. We assume that the noise profile of the microarrays is approximately as that shown in Figure 5e (which seems a typical noise profile for a cell-line experiment - see also Figure 2). The z-score for a differentially expressed gene is:(5)

where  denotes the mean,  denotes the variance of the gene, and *n *denotes the number of repeats in conditions *A*, *B*. Taking *n_A _*= *n_B _*≡ *n *for genes with average expression of 4 and 8 (*σ*^2 ^≈ 0.2, 0.04 respectively), we can predict the minimal fold changes needed to achieve a 3-sigma threshold, for different numbers of repeats.

As can be seen from Table 1, performing more repeats increases the sensitivity, allowing detection of small fold changes as statistically significant, since it is compared to the null hypothesis of identical expression between the conditions. However, a very small fold change, no matter how statistically significant it is, may not be biologically relevant. Therefore, when designing a microarray experiment, we need to take into account the minimal fold change we are interested in detecting, which will affect the number of repeats needed. For example, to detect a minimal fold change of 1.5 at intermediate gene expression (*I *= 8), two repeats at each condition will suffice (see Table 1). Finally, the question of how to distribute the available microarrays for a given experiment is hard to answer. The conflict between having more conditions and having repeats always comes up. Assuming that our assumptions about the noise properties hold, two repeats should suffice to estimate the noise. Whether repeats are needed for all conditions - there is no clear answer but we think the main advantage of having repeats is clearly that they increase our confidence in the results of the analysis.

**Table 1:** Fold change values needed to achieve *p *= 0.0026 (3*σ *deviation), as a function of the number of repeats and the intensity.

	n = 1	n = 2	n = 3
*I *= 4 (*σ *≈ 0.45)	3.73	2.53	2.12

*I *= 8 (*σ *≈ 0.2)	1.8	1.51	1.4

The values of *σ *were taken from the example described in the text.

## Discussion and Conclusions

When using DNA microarrays we are faced with the problem of a small sample size combined with a large number of genes, out of which we want to identify a small differentially expressed subset. Therefore, special care must be taken to correctly estimate the distribution of noise for all the genes. Even a small fraction of genes for which we under-estimate the variance can significantly contaminate the results with false positives. We showed here a method for estimation of the technical noise characteristics of the microarrays. Utilizing the fact that the technical noise is intensity dependent, we are able to use hundreds of genes with similar intensity to get an accurate estimation of the noise profile. Using the resulting estimated variance, we propose a reliable and sensitive method to detect significant changes of expression associated with different biological conditions.

It has been previously shown [9] that the noise level in microarray experiments is low. While being encouraging, this does not mean that this noise can just be ignored. Using a small number of repeats often leads to a noisy estimation of the variance, thus leading to a reduced statistical power when using t-test statistics on each gene separately. This can often lead to non-significant statistics even on genes with a biologically relevant change in expression. On the other hand, as evident from the current study, using the average variance across all genes can lead to false-positives, due to the non-uniformity of variance distribution across genes. By showing that the noise term has approximately normal i.i.d. distribution for genes with similar average intensity, and calculating the intensity-dependent noise profile, one can utilize the low noise levels to accurately identify differentially expressed genes even when the number of samples is small.

## Methods

### Bin size selection

There is no clear criterion to determine the bin size. The selected bin size is the result of a trade-off; on the one hand we wish to have in each bin a large number of points (genes) *N*, in order to improve the statistics (the variance is estimated on the basis of *N *variances measured from the data), while on the other hand one wishes to keep the intensity range that defines the bin small (since genes of similar intensity are hypothesized to have the same variance). In such a case one should examine the sensitivity of the results to the bin size used. We find that having *N *≥ 200 elements in each bin provides a good estimate for the parameters of the desired distribution. In Figure 3 we use a bin width of 0.5 (in *log*_2 _of expression values), which is not optimal, but as can be seen, does provide a distribution very close to normal.

### Empirical probability density function estimation

The experimental probability density functions *f*(*x*) were evaluated by dividing the range of the variable *x *that characterizes the data points (e.g. difference between repeats) to about 30 - 50 equal bins. The number chosen for each case depended on how many data points were available; on the one hand, we want to have small fluctuations of the number of points in a bin, and on the other hand, we wish to have a sufficient number of bins to provide information about the probability density. The counts in each bin were normalized so that the integral of the resulting histogram is 1, and a simple smoothing procedure (running average over 5 elements) was applied.

### Robust variance estimation

The simplest way to estimate the variance of the noise from *n *repeats is to calculate the variance estimator for each gene, and its average over genes with the same intensity. However, such an estimation can be biased by a small number of outlier genes whose variance deviates strongly from the population distribution. Here we provide an iterative procedure to estimate the intensity dependent variance. The procedure uses the assumptions (which were validated before) about the noise distribution, and provides some robustness to exclude outliers. Consider an experiment of *n *repeats, let *I_i,j _*with *j *= 1, 2, , , , *n *be the measured expression levels of gene *i*.

1. Calculate the naive mean and variance estimators  for each gene as described in eq. (1).

2. Define the neighborhood of gene i as all genes k satisfying , where *w *is a chosen bin size (can be fixed or varied).

3. Calculate the intensity dependent variance estimator (IDVE): 

4. Calculate the two tailed p-value for each gene variance estimator () using the IDVE , as follows:(6)

where (*n *- 1) is the number of degrees of freedom and  is the density function. The calculated *p *is the probability that a scaled *χ*^2 ^distributed variable with mean of  will take a value whose deviation from the mean is greater than .

5. Truncate the approximately 1% extreme values of the distribution. This should be done carefully in a way that does not bias the mean for asymmetric distributions (such as chi-square): Define the right cutoff point (outlayers with high variance) by selecting the largest point with *p *> 0.01 (denote it as *x*_2_). To get *x*_1_, the corresponding cutoff value on the left, we solve the integral equation:(7)

These choices of *x*_1 _and *x*_2 _keep the mean at *n *- 1.

6. Repeat the process (steps 2 - 5) with the truncated distribution until it converges (i.e. the IDVE  does not change).

### Datasets used

The data used for this analysis is available on-line from the GEO database. In the first dataset, accession number GSE19921, the RNA measurements (used here as 8 replicates) were done on the breast cancer cell line T47 D, with duplicates taken under the following conditions: wild-type T74 D, shEGFP T47 D and two shRNAs designed to target ERBB3. The 8 microarrays were viewed as replicates since the RNA measurements showed only very minor effects of introducing the shRNA. The second dataset is the MAQC [8], accession number GSE5350, where we used only the Affymetrix arrays from Site 1. The third dataset (accession number GSE17721) from Amit et al [13] is expression data of mouse dendritic cells after LPS stimulation.

### Data preprocessing

Data from the Affymetrix 3' arrays used, HG-U133 Plus 2.0 in [8] and Affymetrix HT-MG-430A in [13], were preprocessed using the Affymetrix MAS5 algorithm and then corrected by a Lowess multi array procedure. The Affymetrix HuGene 1.0 ST array data were preprocessed using the Affymetrix PLIER algorithm (with no normalization) and then corrected by a Lowess multi array procedure.

## Abbreviations

r.v.: random variable; i.i.d.: independent and identical distributed; SD: standard deviation; IDVE: intensity dependent variance estimator; pdf: probability density function; cdf: cumulative distribution function.

## Authors' contributions

AZ, AA and ED participated in the design of the study. AZ and AA performed the statistical analysis and model development and wrote the first draft of the manuscript, which was critically edited by ED, and WK performed the experiment (including qPCR) on the murine dendritic cells. All authors read and approved the final manuscript.

## Supplementary Material

Additional file 1 **Supplemental Information, that contains**: • Modeling the noise as additive and/or multiplicative does not work. • Normality test and sensitivity to bin size. • Microarray noise is independent of GC content. • Z-test versus t-test. • Issues related to biological noise. • Enrichment of differentially expressed genes and PCR validation. Click here for file

Additional file 2 **A MATLAB simulation code for Figure **7. Click here for file
