# Supplementary material for: Intensity dependent estimation of noise in microarrays improves detection of differentially expressed genes
Source: BMC Bioinformatics. 2010 Jul 27;11:400. doi: 10.1186/1471-2105-11-400 (PMC2920277; doi:10.1186/1471-2105-11-400)
Supplement: Additional file 1 — Supplemental Information, that contains: • Modeling the noise as additive and/or multiplicative does not work. • Normality test and sensitivity to bin size. • Microarray noise is independent of GC content. • Z-test versus t-test. • Issues related to biological noise. • Enrichment of differentially expressed genes and PCR validation. [file 1471-2105-11-400-S1.PDF]

Additional file 1: Intensity dependent estimation of noise in microarrays improves detection of differentially expressed genes

Amit Zeisel*, Amnon Amir*, Wolfgang J. Köstler, and Eytan Domany

July 24, 2010

1 Modeling the noise as additive and/or multiplicative does not work

We define technical noise as the variability in intensity of repeats of hybridization of aliquots of the same sample (following RNA extraction) to identical DNA chips. This variability is therefore due to noise in the hybridization and fluorescence signal processing. Since the hybridization for each probe involves a large number of molecules, we assume that noise is not due to discretization effects ($\sigma/\sqrt{n}$). Two typical types of noise which can arise are additive and multiplicative.

1. Additive noise: This noise is expressed as $I = I_0 + U$, (I is signal intensity before *log* transform) where I_0 is the true intensity and U is an intensity *independent* random variable. It may arise for example from variability in the background intensity throughout the chip. This leads to constant absolute noise (as a function of the mean), and a decreasing CV (which is defined as the standard deviation divided by the mean). Following the *log* transformation, the additive noise model will show a decrease in absolute noise as a function of the mean.

2. Multiplicative noise: This noise is expressed as $I = I_0U$, where I_0 is the true intensity and U is an intensity *independent* random variable. It may arise from fluctuations in the illumination intensity of the chip or light reaching the CCD. This leads to an increase in absolute noise level (as a function of the mean) and a constant CV. Following the *log* transformation, multiplicative noise will be of constant level.

In the more general case, we can try to model the noise (before log transform) as a linear combination of these two noise sources: $I = I_0U + V$, where I_0 is the true intensity, and U, V are independent random variables. In this case we expect $Var(I) = I_0^2 Var(U) + Var(V)$. Hence the variance in the measurements should be quadratic in the average intensity. An example of the (non log-transformed) variance as a function of the mean is shown in Figure 1. Panel (a) shows the non log-transformed variance vs. mean scatter plot, with the smoothed variance shown in **magenta**. While it is an increasing function, it is clearly non-quadratic. A good quadratic fit only applies for large mean intensities (**black** line in panel

b), which correspond to only 20% of the genes. The data for the lower intensity genes does not fit well to the model, as can be seen in panel c, therefore indicating that the simple additive and multiplicative noise model does not suffice to describe the origin of the technical noise. Rather than trying to model the noise, we will take for each experiment the measured **intensity - dependent** noise profile.

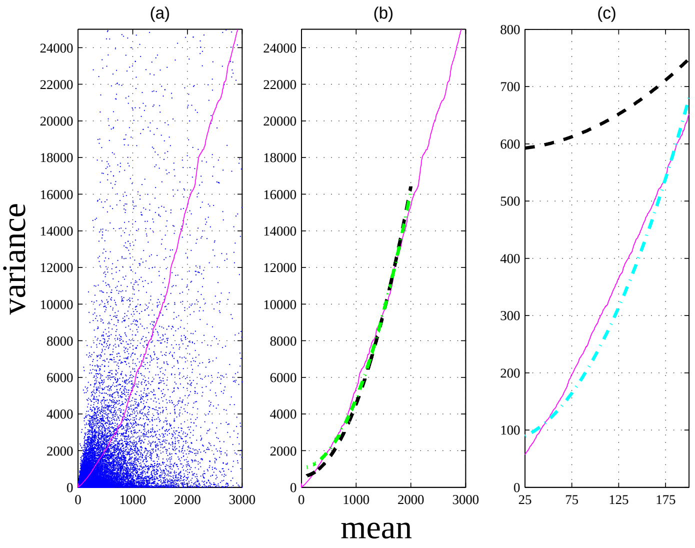

Figure S1: Variance vs. intensity and its fit to a combined additive and multiplicative noise model. (a) scatter of the variance estimator and its running average (magenta line), (b) the fit of the running average line to the combined noise model. The black line was fitted to a larger range of intensities, and the green line to a narrower range (c) zoom in to the fit plot were the black line is the same from panel b and the cyan line is a fit for the low intensity levels only.

2 Normality test and sensitivity to bin size.

We present here tests of normality of the intensity-dependent noise distribution and the effect of the bin size chosen on the results of these tests. The following figures were obtained for the same data (GSE19921) and present the same tests as those shown in Fig 3 of the manuscript.

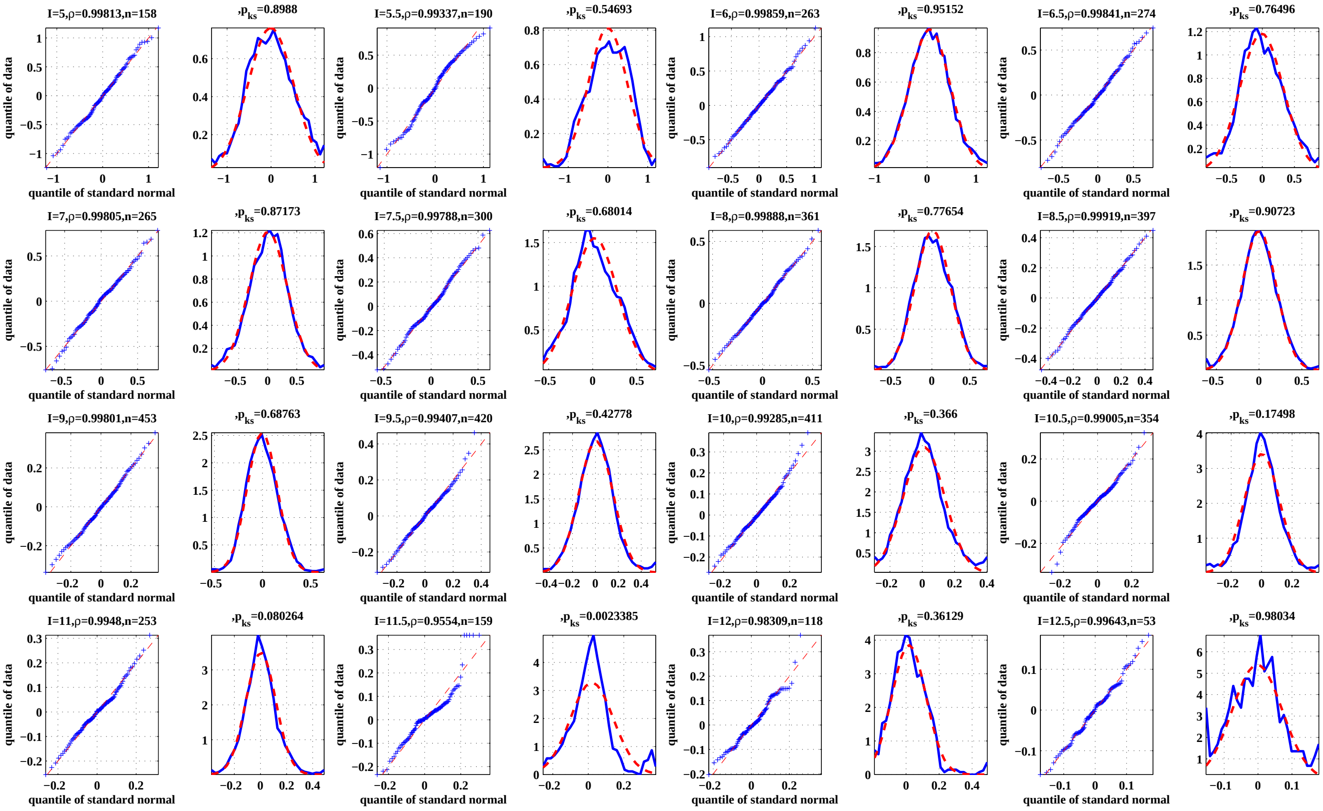

Figure S2: Normality test plots. Q-Q plot and the estimated pdf of the difference between two repeats for a range of intensity bins centered at $5-12.5$, with a bin width of 0.2. For each intensity bin we show on the left the Q-Q plot against the theoretical normal distribution, and on the right the empirical pdf is compared to the theoretical pdf. For each intensity bin the correlation (ρ), the number of elements in the bin (n), and the Kolmogorov-Smirnov p-value (p_{ks}) are indicated.

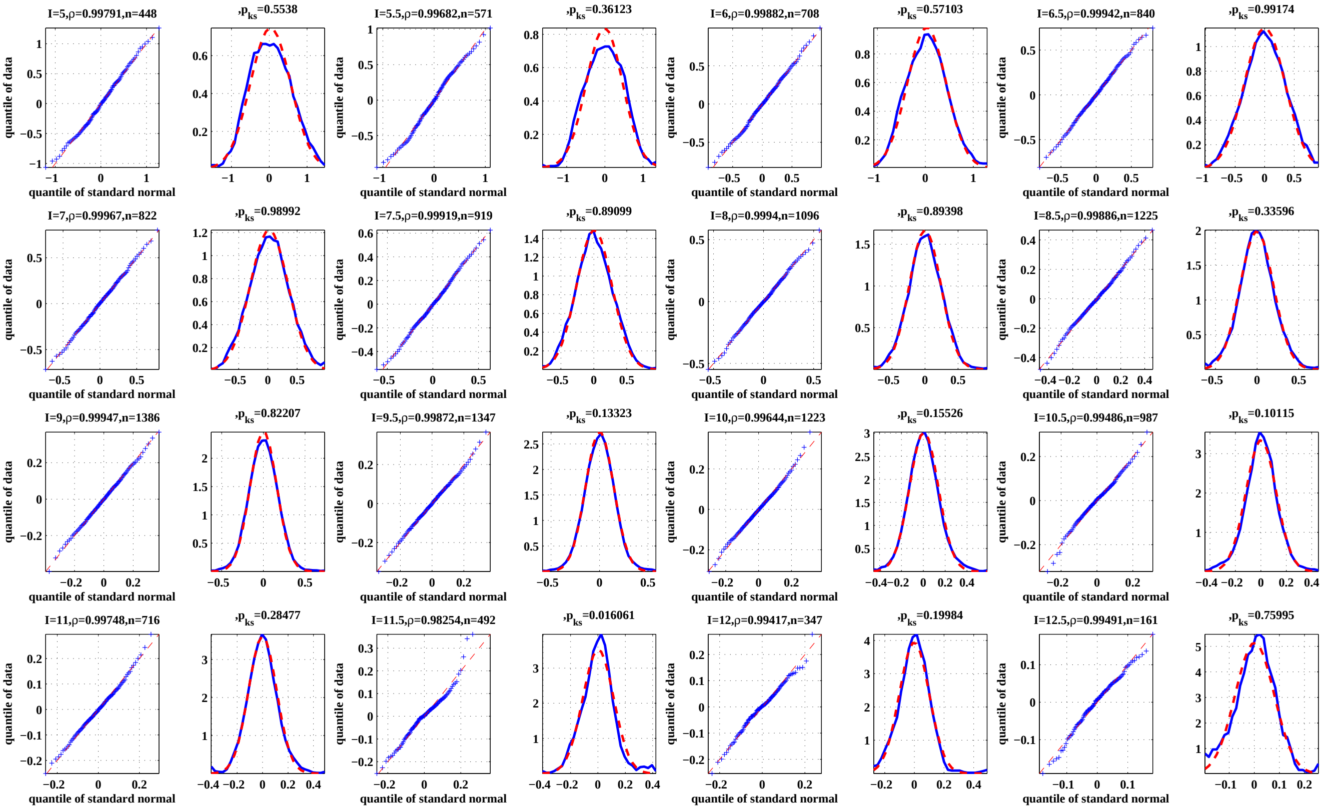

Figure S3: Normality test plots. Q-Q plot and the estimated pdf of the difference between two repeats for a range of intensity bins centered at $5 - 12.5$, with a bin width of 0.6. For each intensity bin we show on the left the Q-Q plot against the theoretical normal distribution, and on the right the empirical pdf is compared to the theoretical pdf. For each intensity bin the correlation (ρ), the number of elements in the bin (n), and the Kolmogorov-Smirnov p-value (p_{ks}) are indicated.

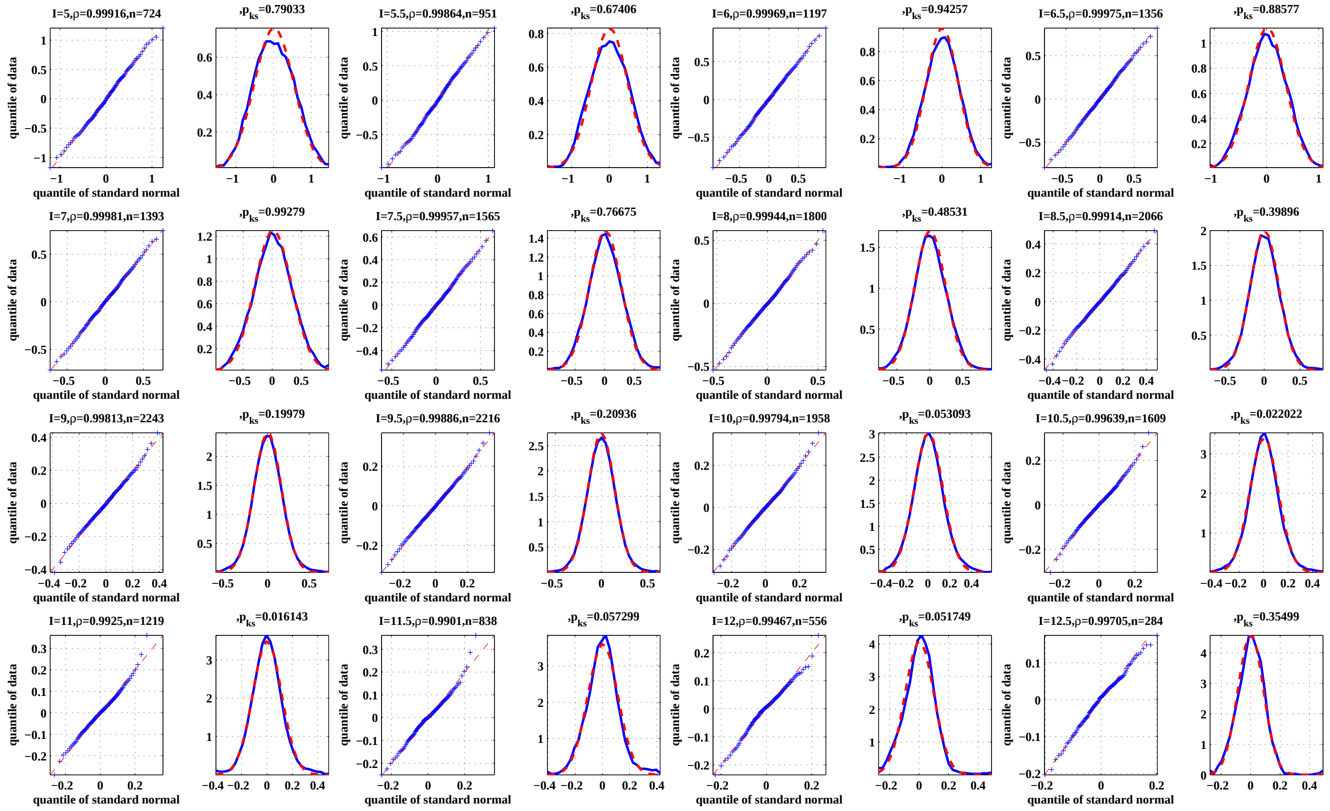

Figure S4: Normality test plots. Q-Q plot and the estimated pdf of the difference between two repeats for a range of intensity bins centered at $5-12.5$, with a bin width of 1. For each intensity bin we show on the left the Q-Q plot against the theoretical normal distribution, and on the right the empirical pdf is compared to the theoretical pdf. For each intensity bin the correlation (ρ), the number of elements in the bin (n), and the Kolmogorov-Smirnov p-value (p_{ks}) are indicated.

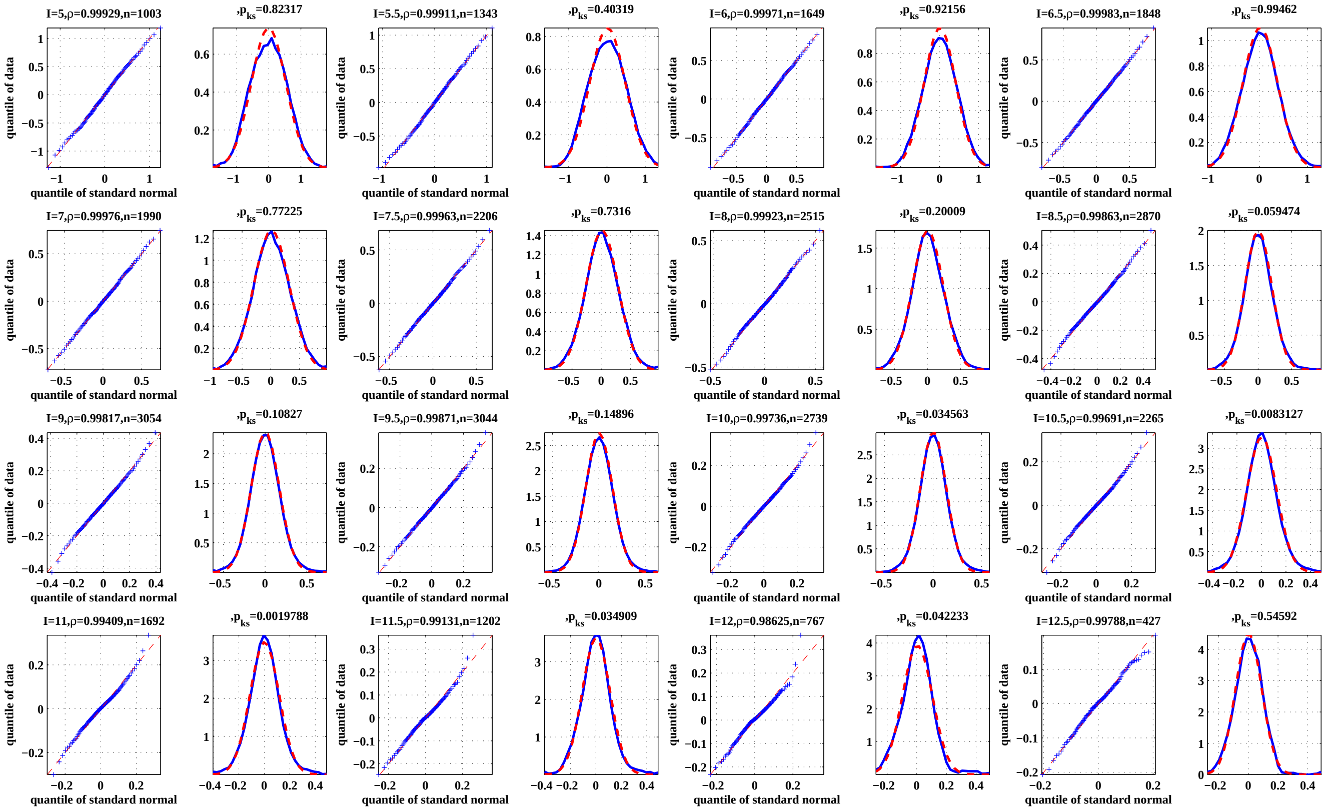

Figure S5: Normality test plots. Q-Q plot and the estimated pdf of the difference between two repeats for a range of intensity bins centered at $5 - 12.5$, with a bin width of 1.4. For each intensity bin we show on the left the Q-Q plot against the theoretical normal distribution, and on the right the empirical pdf is compared to the theoretical pdf. For each intensity bin the correlation (ρ), the number of elements in the bin (n), and the Kolmogorov-Smirnov p-value (p_{ks}) are indicated.

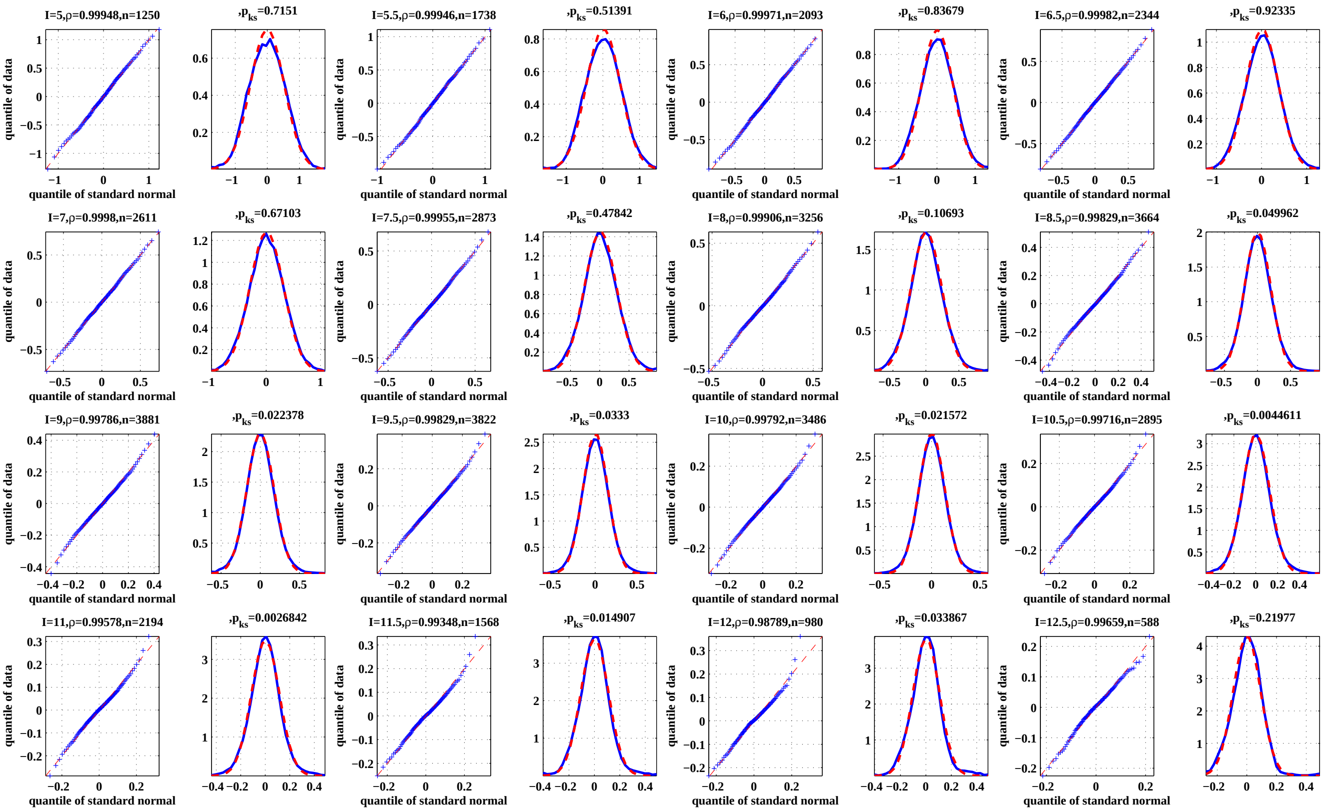

Figure S6: Normality test plots. Q-Q plot and the estimated pdf of the difference between two repeats for a range of intensity bins centered at $5 - 12.5$, with a bin width of 1.8. For each intensity bin we show on the left the Q-Q plot against the theoretical normal distribution, and on the right the empirical pdf is compared to the theoretical pdf. For each intensity bin the correlation (ρ), the number of elements in the bin (n), and the Kolmogorov-Smirnov p-value (p_{ks}) are indicated.

3 Microarray noise is independent of the GC content

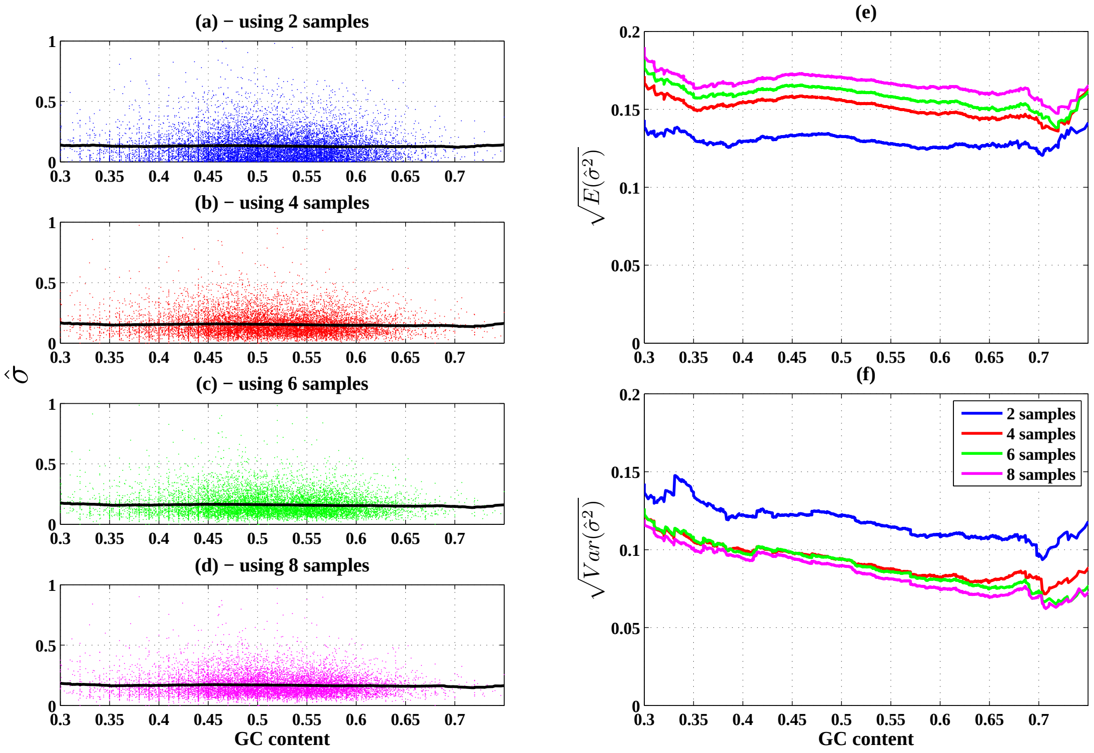

Figure S7: ***Microarray noise is independent of the GC content:*** Scatter plots of the variance square root estimator vs. the GC content of the probe-set and its properties for different numbers of repeats: (a)-(d) show the scatter and the line estimated by our approach (black line), (e) and (f) show the average and standard deviation lines.

4 Z-test versus t-test

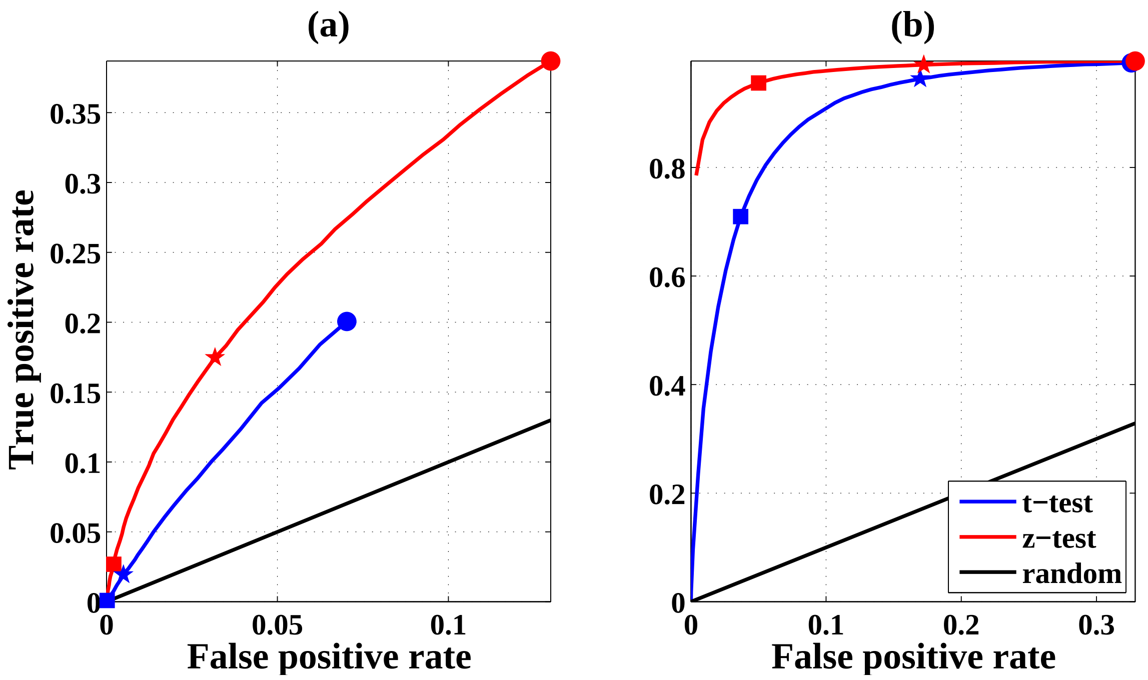

Figure S8: ***Z-test is more powerful than t-test:*** ROC curves demonstrating the superior performance of the z-test over the t-test in identifying differentially expressed genes, on simulated data as described in the main text. (a) - for weak signal (differentially expressed genes with mean difference of 1 standard deviation), (b) - for strong signal (differentially expressed genes with mean difference of 3 standard deviations). The square, star, and circle marks are related to FDR levels of $10, 30, 50\%$ respectively.

5 Issues related to biological noise

Biological noise is defined as the variability observed in a group of samples taken at the same condition, in the ideal case where there is no technical noise. Biological noise can arise from differences in growth conditions (for example cell samples from the same cell line but from different plates), or differences between samples taken from different individuals. This noise reflects the variability in the population rather than a measurement artifact. Biological noise is much harder to detect because it does not have a simple common structure. Unlike in the case of technical noise, we can not assume that intensity dependence suffices to describe biological variation, and hence we can not use the approach described above for estimation of the biological noise. In a real dataset the biological and technical noise are mixed and since we know nothing about the biological noise it is very hard to distinguish between them. The problem in this case remains to accurately estimate the variance. Figure 9 depicts the slow convergence of the naive variance estimator when calculated for each gene separately as can be derived from eq. (3). As can be seen, in order to accurately estimate the variance of each gene separately, a very large number of repeats is needed.

Since the observed variance contains both technical and biological noise, we can claim that the true variance is not smaller than the technical noise alone. We suggest to use our approach in order to provide a lower bound on the variance, by estimating the noise as if had been purely technical. This can be done using the procedures described above.

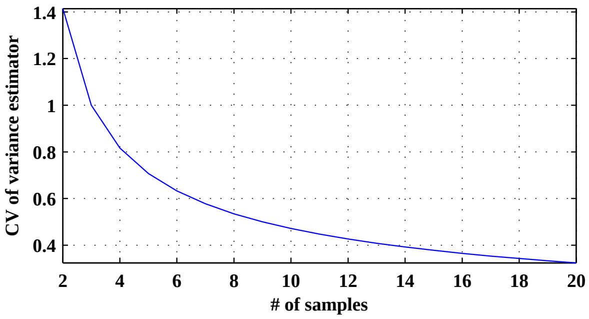

Figure S9: ***Slow convergence of the naive variance estimator:*** Coefficient of variance (ratio of standard deviation to mean) for the variance estimator vs. the number of samples, as expected from normally distributed samples, see eq. (3).

6 Enrichment of differentially expressed genes and PCR validation

Differentially expressed genes in pairs of consecutive time points of the data of Amit et al. [1] were detected using our method (z-test) as described in the main text. The resulting gene lists were analyzed for functional enrichment using the DAVID web tool [2] using the Affymetrix Mouse Genome 430A 2.0 Array as background. Enrichment analysis was performed for gene ontology terms, tissue expression, protein domains, functional categories and pathways. Shown are terms which contain at least 20 genes and with a fold enrichment of at least 2, and are sorted in descending order according to fold enrichment.

Abbreviations used are: GO - gene ontology, PW - pathway, TIS - tissue, FC - functional category, PD - protein domain.

0.5 hr vs. 1 hr	1 hr vs. 2 hr	2 hr vs. 4 hr
PW:Toll-like receptor signaling pathway	GO:regulation of cytokine production	GO:early endosome
TIS:Macrophage	PW:Toll-like receptor signaling pathway	PW:Apoptosis
GO:inflammatory response	GO:regulation of mononuclear cell proliferation	GO:nuclear import
FC:Apoptosis	GO:regulation of lymphocyte proliferation	PW:Chronic myeloid leukemia
GO:leukocyte activation	FC:electron transport	GO:chromatin remodeling complex
GO:hemopoiesis	GO:regulation of leukocyte proliferation	GO:protein import into nucleus
GO:cell activation	GO:electron transport chain	GO:protein localization in nucleus
TIS:Bone marrow	GO:negative regulation of catalytic activity	TIS:Bone marrow
TIS:Mast cell	GO:positive regulation of lymphocyte activation	GO:cytokine-mediated signaling pathway
GO:hemopoietic or lymphoid organ development	GO:positive regulation of cell activation	TIS:Macrophage
GO:immune response	GO:positive regulation of leukocyte activation	FC:nucleotidyltransferase
PD:Nucleotide-binding, alpha-beta plait	GO:ribosome	
GO:cell proliferation	TIS:Macrophage	
GO:membrane invagination	TIS:Dendritic cell	
GO:endocytosis	FC: compositionally biased region:Gln-rich	
GO:protein domain specific binding		
GO:response to wounding		
GO:defense response		
GO:small GTPase mediated signal transduction		
GO:immune system development		
TIS:Activated spleen		
GO:blood vessel morphogenesis		
FC:methylation		
GO:regulation of cell cycle		
GO:internal side of plasma membrane		
PW:Chemokine signaling pathway		
GO:positive regulation of immune system process		
FC:nucleotide phosphate-binding region:GTP		

Table S1: Enriched GO terms for genes identify to be differentially expressed by our method when comparing: 0.5 hr vs. 1 hr, 1 hr vs. 2 hr, 2 hr vs. 4 hr.

References

[1] Ido Amit, Manuel Garber, Nicolas Chevrier, Ana Paula Leite, Yoni Donner, Thomas Eisenhaure, Mitchell Guttman, Jennifer K. Grenier, Weibo Li, Or Zuk, Lisa A. Schubert, Brian Birditt, Tal Shay, Alon Goren, Xiaolan Zhang, Zachary Smith, Raquel Deering, Rebecca C. McDonald, Moran Cabili, Bradley E. Bernstein, John L. Rinn, Alex Meissner, David E. Root, Nir Hacohen, and Aviv Regev. Unbiased reconstruction of a mammalian transcriptional network mediating pathogen responses. *Science*, 326:257–263, 2009.

4 hr vs. 6 hr	6 hr vs. 8 hr	8 hr vs. 12 hr
PW:Fc gamma R-mediated phagocytosis	GO:response to wounding	GO:positive regulation of cytokine production
TIS:Amnion	TIS:Macrophage	GO:regulation of cytokine production
GO:leukocyte differentiation	TIS:Amnion	GO:protein folding
FC:prenylation	GO:defense response	TIS:Amnion
GO:membrane invagination	GO:cellular response to stress	GO:cell cortex
GO:endocytosis	TIS:Activated spleen	TIS:Macrophage
TIS:Macrophage		TIS:Bone marrow
TIS:Bone marrow		GO:pigment granule
FC:isopeptide bond		GO:melanosome
GO:membrane organization		TIS:Mammary tumor. Brca1-/fl
GO:monosaccharide metabolic process		GO:positive regulation of cell activation
GO:generation of precursor metabolites and energy		GO:electron transport chain
GO:hexose metabolic process		GO:positive regulation of leukocyte activation
GO:protein domain specific binding		
GO:leukocyte activation		
GO:lysosome		
GO:lytic vacuole		

Table S2: Enriched GO terms for genes identified as differentially expressed by our method when comparing: 4 hr vs. 6 hr, 6 hr vs. 8 hr, 8 hr vs. 12 hr.

12 hr vs. 16 hr	16 hr vs. 24 hr
FC:respiratory chain	FC:immune response
GO:respiratory chain	GO:immune effector process
FC:electron transport	GO:regulation of cytokine production
GO:regulation of mononuclear cell proliferation	GO:immune response
GO:regulation of lymphocyte proliferation	GO:regulation of cell activation
GO:regulation of leukocyte proliferation	GO:inflammatory response
GO:electron transport chain	GO:positive regulation of response to stimulus
FC:inflammatory response	GO:positive regulation of immune response
GO:positive regulation of lymphocyte activation	GO:regulation of lymphocyte activation
FC:lysosome	GO:regulation of leukocyte activation
GO:positive regulation of cell activation	GO:cell activation
GO:positive regulation of leukocyte activation	TIS:Bone marrow
PW:NOD-like receptor signaling pathway	GO:leukocyte activation
TIS:B-cell	GO:positive regulation of immune system process
PW:Toll-like receptor signaling pathway	GO:lymphocyte activation
TIS:Bone marrow	GO:response to wounding
FC:immune response	GO:cell surface
GO:adaptive immune response	GO:lysosome
PW:Systemic lupus erythematosus	GO:positive regulation of signal transduction
TIS:Ubiquitous.	TIS:Macrophage
PW:B cell receptor signaling pathway	GO:defense response
TIS:Macrophage	GO:external side of plasma membrane
GO:generation of precursor metabolites and energy	GO:positive regulation of cell communication
TIS:Mammary tumor. Brca1-/fl	GO:vacuole
GO:melanosome	PW:Chemokine signaling pathway
GO:pigment granule	GO:cell proliferation
GO:lysosome	TIS:Activated spleen
GO:lytic vacuole	GO:enzyme activator activity
GO:positive regulation of immune system process	TIS:Mast cell
	GO:locomotory behavior

Table S3: Enriched GO terms for genes identified as differentially expressed by our method when comparing: 12 hr vs. 16 hr, 16 hr vs. 24 hr.

[2] Da Wei Huang, Brad T. Sherman, and Richard A. Lempicki. Systematic and integrative analysis of large gene lists using DAVID bioinformatics resources. *Nat Protoc*, 4:44–57, 2009.

0.5 hr vs. 1 hr	1 hr vs. 2 hr	2 hr vs. 4 hr
GO:guanyl nucleotide binding	FC:electron transport	FC:RRM 1
GO:guanyl ribonucleotide binding	GO:electron transport chain	FC:RRM 2
GO:vesicle-mediated transport	PW:Parkinson's disease	TIS:B-cell
GO:GTP binding	ribosomal protein	PD:RRM
GO:cytosol	GO:structural constituent of ribosome	PD:RNA recognition motif, RNP-1
FC:Apoptosis	FC:mitochondrion inner membrane	GO:protein targeting
TIS:Mammary tumor. C3	PW:Oxidative phosphorylation	PD:Nucleotide-binding, alpha-beta plait
GO:immune system development	GO:generation of precursor metabolites and energy	TIS:Bone marrow
FC:gtp-binding	GO:ribosome	GO:protein folding
GO:hemopoietic or lymphoid organ development	FC:ribonucleoprotein	FC:methylation
FC:nucleotide phosphate-binding region:GTP	PW:Huntington's disease	GO:spliceosome
GO:cell proliferation	GO:positive regulation of immune system process	TIS:Amnion
GO:hemopoiesis	GO:translation	GO:intracellular protein transport
GO:cell activation	GO:mitochondrial inner membrane	TIS:Macrophage
TIS:Bone marrow	PD:RRM	TIS:Mast cell
GO:small GTPase mediated signal transduction	PW:Alzheimer's disease	GO:cellular protein localization
TIS:Macrophage	GO:organelle inner membrane	GO:cellular macromolecule localization
	GO:mitochondrial part	FC:protein biosynthesis
	PD:RNA recognition motif, RNP-1	FC:mrna splicing
	FC:isopeptide bond	FC:rna-binding
	GO:positive regulation of response to stimulus	GO:RNA splicing
	GO:mitochondrial envelope	PW:Oxidative phosphorylation
	GO:mitochondrial membrane	GO:generation of precursor metabolites and energy
	PD:Nucleotide-binding, alpha-beta plait	FC:mrna processing
	GO:ribonucleoprotein complex	GO:intracellular transport
	TIS:Macrophage	FC:Chaperone
	FC:mitochondrion	FC:acetylation
	GO:organelle envelope	FC:isopeptide bond
	GO:envelope	GO:mRNA processing
	FC:rna-binding	GO:cellular macromolecular complex assembly

Table S4: Enriched GO terms for genes identified as differentially expressed uniquely by our method (not identified by either SAM, t-test or 2fold change) when comparing: 0.5 hr vs. 1 hr, 1 hr vs. 2 hr, 2 hr vs. 4 hr.

4 hr vs. 6 hr	6 hr vs. 8 hr	8 hr vs. 12 hr
PW:Fc gamma R-mediated phagocytosis	GO:response to wounding	GO:regulation of cytokine production
TIS:Amnion	TIS:Amnion	GO:protein folding
FC:prenylation	TIS:Macrophage	TIS:Amnion
GO:leukocyte differentiation	GO:defense response	TIS:Macrophage
TIS:Macrophage	TIS:Activated spleen	TIS:Bone marrow
GO:endocytosis	GO:cellular response to stress	PD:RRM
GO:membrane invagination		FC:Chaperone
TIS:Bone marrow		GO:nuclear envelope
GO:monosaccharide metabolic process		GO:immune effector process
FC:isopeptide bond		PW:Parkinson's disease
GO:protein domain specific binding		FC:ribosomal protein
GO:generation of precursor metabolites and energy		FC:immune response
GO:hexose metabolic process		GO:protein domain specific binding
GO:lytic vacuole		PW:Nucleotide-binding, alpha-beta plait
GO:lysosome		
GO:membrane organization		
FC:immune response		

Table S5: Enriched GO terms for genes identified as differentially expressed uniquely by our method (not identified by either SAM, t-test or 2fold change), when comparing: 4 hr vs. 6 hr, 6 hr vs. 8 hr, 8 hr vs. 12 hr.

12 hr vs. 16 hr	16 hr vs. 24 hr
FC:electron transport	GO:regulation of cell activation
TIS:B-cell	FC:immune response
GO:electron transport chain	GO:regulation of lymphocyte activation
TIS:Mammary tumor. Brca1-/fl	GO:regulation of leukocyte activation
PW:B cell receptor signaling pathway	GO:immune response
FC:lysosome	GO:cell activation
TIS:Bone marrow	TIS:Bone marrow
FC:mitochondrion inner membrane	GO:leukocyte activation
FC:immune response	GO:lymphocyte activation
PW:Parkinson's disease	GO:positive regulation of response to stimulus
GO:cell leading edge	GO:lytic vacuole
TIS:Macrophage	GO:lysosome
PW:Toll-like receptor signaling pathway	GO:cell surface
TIS:Amnion	GO:external side of plasma membrane
PW:Fc gamma R-mediated phagocytosis	GO:inflammatory response
GO:generation of precursor metabolites and energy	GO:positive regulation of immune system process
PW:Huntington's disease	TIS:Macrophage
GO:glucose metabolic process	GO:positive regulation of signal transduction
TIS:Dendritic cell	GO:vacuole
GO:GTPase activity	GO:positive regulation of cell communication
PD:Small GTP-binding protein	GO:response to wounding
GO:vacuole	TIS:Mast cell
	GO:defense response
	PW:Chemokine signaling pathway
	GO:cell proliferation
	GO:phosphatase activity

Table S6: Enriched GO terms for genes identified as differentially expressed uniquely by our method (not identified by either SAM, t-test or 2fold change), when comparing: 12 hr vs. 16 hr, 16 hr vs. 24 hr.

1hr vs. 2hr	2hr vs. 4hr	12hr vs. 16hr
GO:nuclear lumen	GO:DNA replication	GO:phosphorus metabolic process
GO:DNA binding	GO:transcription, DNA-dependent	GO:phosphate metabolic process
FC:Transcription	GO:ncRNA processing	FC:nucleotide-binding
GO:transcription	GO:RNA biosynthetic process	
GO:intracellular organelle lumen	GO:chromosome	
GO:organelle lumen	GO:chromosomal part	
GO:membrane-enclosed lumen	FC:cell division	
	GO:ncRNA metabolic process	
	GO:cell division	
	FC:DNA damage	
	GO:DNA metabolic process	
	FC:mitosis	
	GO:mitotic cell cycle	
	GO:protein complex biogenesis	
	GO:protein complex assembly	
	PD:Zinc finger, C2H2-type/integrase, DNA-binding	
	GO:DNA repair	
	FC:zinc finger region:C2H2-type 1	
	GO:mitosis	
	GO:nuclear division	
	GO:organelle fission	
	GO:M phase of mitotic cell cycle	
	GO:response to DNA damage stimulus	
	FC:cell cycle	
	PD:ZnF-C2H2	
	PD:Zinc finger, C2H2-like	
	PD:Zinc finger, C2H2-type	
	GO:nuclear lumen	
	GO:nucleoplasm	
	GO:macromolecular complex assembly	
	GO:nucleolus	
	FC:zinc finger region:C2H2-type 2	
	GO:intracellular organelle lumen	
	GO:organelle lumen	
	GO:membrane-enclosed lumen	
	GO:nucleoplasm part	
	GO:cell cycle phase	

Table S7: Enriched GO terms for genes identified as differentially expressed uniquely by SAM (not identified by either 2fold or our method) when comparing: 1 hr vs. 2hr, 2 hr vs. 4 hr, 12 hr vs. 16 hr.

0.5 hr vs. 1 hr	4 hr vs. 6 hr	6 hr vs. 8 hr
GO:intracellular signaling cascade	FC:transferase	FC:zinc-finger
FC:zinc	FC:hydrolase	FC:transferase
TIS:Thymus	TIS:Thymus	FC:zinc
FC:nucleotide-binding	FC:metal-binding	GO:zinc ion binding
GO:zinc ion binding	GO:transition metal ion binding	TIS:Thymus
GO:transcription	GO:cation binding	GO:transition metal ion binding
FC:Transcription	GO:ion binding	FC:metal-binding
FC:metal-binding		GO:metal ion binding
GO:purine ribonucleotide binding		GO:cation binding
GO:ribonucleotide binding		GO:ion binding
TIS:Testis		FC:phosphoprotein
TIS:Brain		
FC:nucleus		

Table S8: Enriched GO terms for genes identified as differentially expressed uniquely by 2 fold threshold (not identified either by SAM or our method) when comparing: 0.5 hr vs. 1 hr, 4 hr vs. 6 hr, 6 hr vs. 8 hr.

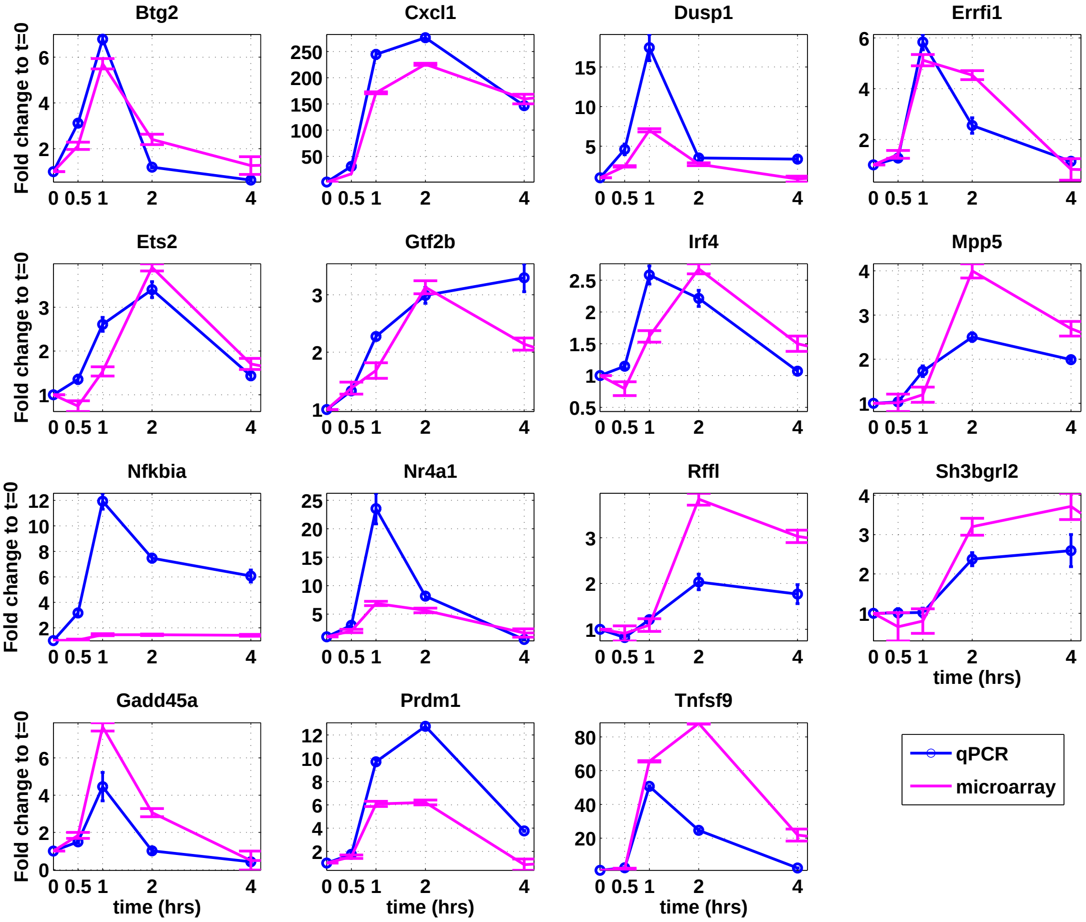

Figure S10: ***Comparison between microarray and qPCR profiles for the data of Amit et al:*** The profiles of 15 selected genes, identified as differentially expressed by our method (when comparing: 0.5 hr vs. 1 hr, 1 hr vs. 2 hr, 2 hr vs. 4 hr) were compared to qPCR profiles obtained by us in an independently performed experiment. The genes Rffl, Sh3bgrl2, Gadd45a, Pdrm1, Tnfsf9, which were not identified by the SAM method or t-test as significantly varying between time points, in fact do exhibit variation. Note that errorbars in the qPCR curves represent standard deviation for three technical repeats, while in the microarray curves errorbars indicate standard deviation as estimated by our method.
