# Supplementary material for: Intensity dependent estimation of noise in microarrays improves detection of differentially expressed genes
Source: BMC Bioinformatics. 2010 Jul 27;11:400. doi: 10.1186/1471-2105-11-400 (PMC2920277; doi:10.1186/1471-2105-11-400)
Supplement: Additional file 2 — A MATLAB simulation code for Figure 7. [file 1471-2105-11-400-S2.PDF]

```
tic
close all
clear all


N=1000;
k=50;
m=100;
v=[true(1,N/2),false(1,N/2)];
tp_tt=zeros(1,k);
fp_tt=zeros(1,k);
tp_z=zeros(1,k);
fp_z=zeros(1,k);
Rtt=zeros(1,k);
Rz=zeros(1,k);
for i=1:m
    i
    x1=normrnd(0,1,N,3);
    x2=[normrnd(1,1,N/2,3);normrnd(0,1,N/2,3)];

    [h,ptt]=ttest2(x1',x2');
    pz=2*normcdf(-abs(mean(x1,2)-mean(x2,2)),0,sqrt(2/3));
    for j=1:k
        ftt=fdr_proc(ptt,j/m);
        fz=fdr_proc(pz,j/m);
        Rtt(j)=Rtt(j)+length(ftt);
        Rz(j)=Rz(j)+length(fz);
        tp_tt(j)=tp_tt(j)+sum(v(ftt))/N*2;
        tp_z(j)=tp_z(j)+sum(v(fz))/N*2;
        fp_tt(j)=fp_tt(j)+sum(~v(ftt))/N*2;
        fp_z(j)=fp_z(j)+sum(~v(fz))/N*2;
    end
end
tp_tt=tp_tt/m;
fp_tt=fp_tt/m;
tp_z=tp_z/m;
fp_z=fp_z/m;
Rz=Rz/m;
Rtt=Rtt/m;

figure(1);
set(gcf,'color','w','position',[200,200,900,450])
subplot(1,2,1)
plot(fp_tt,tp_tt,'linewidth',2);grid on; hold on;
plot(fp_z,tp_z,'r','linewidth',2);grid on; hold on;
plot([0,fp_z(end)],[0,fp_z(end)],'k','linewidth',2);
d=10;
plot(fp_tt(d),tp_tt(d),'s','markersize',10,'markerfacecolor','b');grid on; hold on
plot(fp_z(d),tp_z(d),'sr','markersize',10,'markerfacecolor','r');grid on; hold on;
d=30;
plot(fp_tt(d),tp_tt(d),'p','markersize',10,'markerfacecolor','b');grid on; hold on
plot(fp_z(d),tp_z(d),'pr','markersize',10,'markerfacecolor','r');grid on; hold on;
d=50;
plot(fp_tt(d),tp_tt(d),'o','markersize',10,'markerfacecolor','b');grid on; hold on
plot(fp_z(d),tp_z(d),'or','markersize',10,'markerfacecolor','r');grid on; hold on;
set(gca,'fontname','times','fontweight','demi','fontsize',16)
ylabel('True positive rate','fontsize',20);
xlabel('False positive rate','fontsize',20);
title('(a)','fontsize',20)
axis tight
figure(2);
set(gcf,'color','w','position',[200,200,900,450])
subplot(1,2,1)
```

```
plot([1:k],tp_tt,'linewidth',2);grid on; hold on;
plot([1:k],tp_z,'r','linewidth',2);grid on; hold on;
set(gca,'fontname','times','fontweight','demi','fontsize',16)
title('(a)','fontsize',20)
ylabel('True positive rate','fontsize',20);
xlabel('BH FDR(%)','fontsize',20);
legend('t-test','z-test',2)
subplot(1,2,2)
plot([1:k],fp_tt,'linewidth',2);grid on; hold on;
plot([1:k],fp_z,'r','linewidth',2);grid on; hold on;
set(gca,'fontname','times','fontweight','demi','fontsize',16)
title('(b)','fontsize',20)
ylabel('False positive rate','fontsize',20);
xlabel('BH FDR(%)','fontsize',20);


tp_tt=zeros(1,k);
fp_tt=zeros(1,k);
tp_z=zeros(1,k);
fp_z=zeros(1,k);
for i=1:m
    i
    x1=normrnd(0,1,N,3);
    x2=[normrnd(3,1,N/2,3);normrnd(0,1,N/2,3)];

    [h,ptt]=ttest2(x1',x2');
    pz=2*normcdf(-abs(mean(x1,2)-mean(x2,2)),0,sqrt(2/3));
    for j=1:k
        ftt=fdr_proc(ptt,j/m);
        fz=fdr_proc(pz,j/m);
        tp_tt(j)=tp_tt(j)+sum(v(ftt))/N*2;
        tp_z(j)=tp_z(j)+sum(v(fz))/N*2;
        fp_tt(j)=fp_tt(j)+sum(~v(ftt))/N*2;
        fp_z(j)=fp_z(j)+sum(~v(fz))/N*2;
    end
end
tp_tt=tp_tt/m;
fp_tt=fp_tt/m;
tp_z=tp_z/m;
fp_z=fp_z/m;

figure(1);
subplot(1,2,2)
plot(fp_tt,tp_tt,'linewidth',2);grid on; hold on;
plot(fp_z,tp_z,'r','linewidth',2);grid on; hold on;
plot([0,fp_z(end)],[0,fp_z(end)],'k','linewidth',2);
d=10;
plot(fp_tt(d),tp_tt(d),'s','markersize',10,'markerfacecolor','b');grid on; hold on
plot(fp_z(d),tp_z(d),'sr','markersize',10,'markerfacecolor','r');grid on; hold on;
d=30;
plot(fp_tt(d),tp_tt(d),'p','markersize',10,'markerfacecolor','b');grid on; hold on
plot(fp_z(d),tp_z(d),'pr','markersize',10,'markerfacecolor','r');grid on; hold on;
d=50;
plot(fp_tt(d),tp_tt(d),'o','markersize',10,'markerfacecolor','b');grid on; hold on
plot(fp_z(d),tp_z(d),'or','markersize',10,'markerfacecolor','r');grid on; hold on;
set(gca,'fontname','times','fontweight','demi','fontsize',16)
xlabel('False positive rate','fontsize',20);
title('(b)','fontsize',20)
axis tight
legend('t-test','z-test','random',4)
% subplot(2,2,4)
% plot([1:k],tp_tt,'linewidth',2);grid on; hold on;
% plot([1:k],tp_z,'r','linewidth',2);grid on; hold on;
% set(gca,'fontname','times','fontweight','demi','fontsize',16)
% title('(d)','fontsize',20)
```

```
% xlabel('BH FDR(%)','fontsize',20);

toc

i =

     1

i =

     2

i =

     3

i =

     4

i =

     5

i =

     6

i =

     7

i =

     8

i =

     9

i =

    10

i =

    11

i =
```

12

i =

13

i =

14

i =

15

i =

16

i =

17

i =

18

i =

19

i =

20

i =

21

i =

22

i =

23

i =

24

i =

25

i =

26

i =

27

i =

28

i =

29

i =

30

i =

31

i =

32

i =

33

i =

34

i =

35

i =

36

i =

37

i =

38

i =

39

i =

40

i =

41

i =

42

i =

43

i =

44

i =

45

i =

46

i =

47

i =

48

i =

49

i =

50

```
i =
    51
i =
    52
i =
    53
i =
    54
i =
    55
i =
    56
i =
    57
i =
    58
i =
    59
i =
    60
i =
    61
i =
    62
i =
    63
```

i =

64

i =

65

i =

66

i =

67

i =

68

i =

69

i =

70

i =

71

i =

72

i =

73

i =

74

i =

75

i =

76

i =

77

i =

78

i =

79

i =

80

i =

81

i =

82

i =

83

i =

84

i =

85

i =

86

i =

87

i =

88

i =

```
    89
i =
    90
i =
    91
i =
    92
i =
    93
i =
    94
i =
    95
i =
    96
i =
    97
i =
    98
i =
    99
i =
   100
i =
     1
```

```
i =
      2
i =
      3
i =
      4
i =
      5
i =
      6
i =
      7
i =
      8
i =
      9
i =
     10
i =
     11
i =
     12
i =
     13
i =
     14
```

i =

15

i =

16

i =

17

i =

18

i =

19

i =

20

i =

21

i =

22

i =

23

i =

24

i =

25

i =

26

i =

27

i =

28

i =

29

i =

30

i =

31

i =

32

i =

33

i =

34

i =

35

i =

36

i =

37

i =

38

i =

39

i =

40

i =

41

i =

42

i =

43

i =

44

i =

45

i =

46

i =

47

i =

48

i =

49

i =

50

i =

51

i =

52

i =

53

i =

54

i =

55

i =

56

i =

57

i =

58

i =

59

i =

60

i =

61

i =

62

i =

63

i =

64

i =

65

i =

66

i =

67

i =

68

i =

69

i =

70

i =

71

i =

72

i =

73

i =

74

i =

75

i =

76

i =

77

i =

78

i =

79

i =

80

i =

81

i =

82

i =

83

i =

84

i =

85

i =

86

i =

87

i =

88

i =

89

i =

90

i =

91

```
i =

    92

i =

    93

i =

    94

i =

    95

i =

    96

i =

    97

i =

    98

i =

    99

i =

   100

Elapsed time is 11.092903 seconds.
```

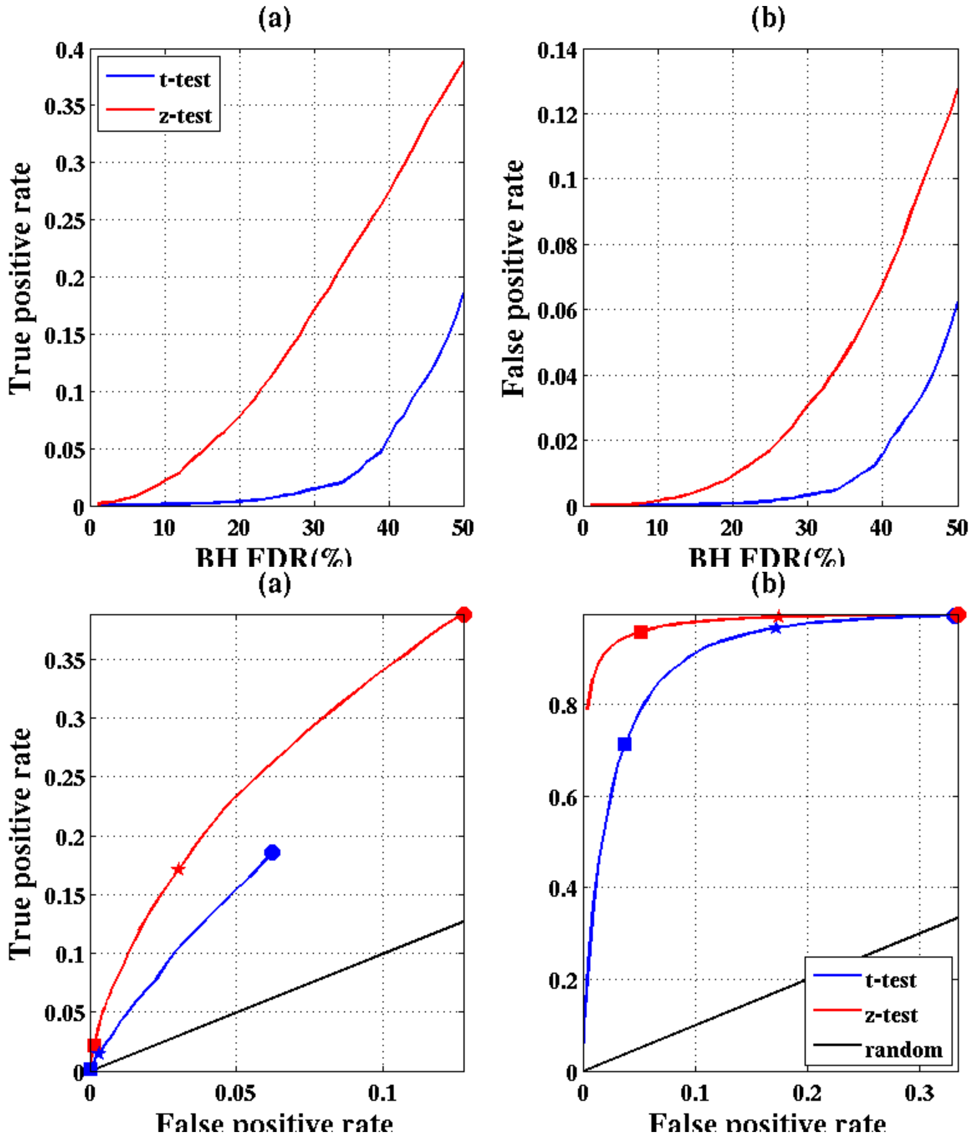

Published with MATLAB® 7.10
